# Supplementary material for: Development of high-resolution multiple-SNP arrays for genetic analyses and molecular breeding through genotyping by target sequencing and liquid chip
Source: Plant Commun. 2021 Aug 9;2(6):100230. doi: 10.1016/j.xplc.2021.100230 (PMC8577115; doi:10.1016/j.xplc.2021.100230)
Supplement: Document S1. Supplemental Figures 1–7, supplemental Tables 1–5, and supplemental note [file mmc1.pdf]

Plant Communications, Volume *2*

Supplemental information

Development of high-resolution multiple-SNP arrays for genetic analyses and molecular breeding through genotyping by target sequencing and liquid chip

Zifeng Guo, Quannv Yang, Feifei Huang, Hongjian Zheng, Zhiqin Sang, Yanfen Xu, Cong Zhang, Kunsheng Wu, Jiajun Tao, Boddupalli M. Prasanna, Michael S. Olsen, Yunbo Wang, Jianan Zhang, and Yunbi Xu

Supplemental Text. The script used for calling mSNPs from each amplicon

1. Quality Control

fastp -i 2019B97_1.fq.gz -I 2019B97__2.fq.gz -o 2019B97_clean_1.fq.gz -O 2019B97_clean_2.fq.gz -h 2019B97.fastp.html -j 2019B97.fastp.json -n 10 -q 20 -u 40 2> 2019B97.fastp.log

2. Data Mapping

bwa mem -t 5 -R "@RG\tID:2019B94\tSM:2019B94\tPL:ILLUMINA" Zea_mays.AGPv3.31.dna.genome.fa 2019B97_clean_1.fq.gz 2019B97_clean_2.fq.gz | samtools view -b -S -T Zea_mays.AGPv3.31.dna.genome.fa - > 2019B94.bam

3. Filter After Mapping

samtools view -h 2019B97.bam |awk '{if($1~/@/){print}else{if($7 =="=" && $5>=30){print $0}}}' |samtoolsview -b -S -T Zea_mays.AGPv3.31.dna.genome.fa - > 2019B97.bf.bam

4. SNP discovery

java -Djava.io.tmpdir=/home/tmp -Xmx15g -jar picard.jar SortSam I=2019B97.bf.bam O=2019B97.sort.bam SORT_ORDER=coordinate VALIDATION_STRINGENCY=LENIENT
java -Djava.io.tmpdir=/home/tmp -Xmx15g-jarpicard.jar MarkDuplicates INPUT=2019B97.sort.bam OUTPUT=2019B97.sort.mdup.bam METRICS_FILE=2019B97.sort.mdup.metrics CREATE_INDEX=true VALIDATION_STRINGENCY=LENIENT
samtools index 2019B97.sort.bam
bedtools intersect -a 2019B97.sort.bam -b insert.bed >2019B97.sort.target.bam
samtools index 2019B97.sort.target.bam
java -Djava.io.tmpdir=/home/tmp -Xmx12g -jar GenomeAnalysisTK.jar -T UnifiedGenotyper -dcov 1000000 -nt 5 -minIndelFrac 0.15 -glm BOTH -l INFO -R Zea_mays.AGPv3.31.dna.genome.fa -I 2019B97.sort.target.bam -o 2019B97.vcf

5. SNP filter

*java -Xmx12g -jar GenomeAnalysisTK.jar -T VariantFiltration -R Zea_mays.AGPv3.31.dna.genome.fa -o 2019B97.filt.vcf –variant 2019B97.vcf --filterExpression "MQ0 >= 4 && ((MQ0 / (1.0 * DP)) > 0.1) " --filterName "HARD_TO_VALIDATE" --filterExpression "DP < 5 || QD < 2" --filterName "LOW_READ_SUPPORT"*

Supplemental Tables

Supplemental Table 1. Multiple target mSNP panels (1K to 40K) generated from 40K mSNP mother panel and their high-PIC SNPs, random SNPs, total SNPs and haplotypes, evaluated with the marker data of 867 maize germplasm accessions

SNPs derived from 40K mSNPs		40K	20K	10K	5K	2K	1K
**High-PIC SNPs**	Marker #	38,034	21,677	10,085	5,000	2,000	1,000
	MAF>5%	33,080	18,745	8,702	4,389	1,769	884
**Random SNPs**	Marker #	38,034	21,677	10,085	5,000	2,000	1,000
	MAF>5%	35,837	20,437	9,488	4726	1,893	947
**Total SNPs**	Marker #	251,630	148,936	69,327	33,711	13,101	6,493
	MAF>5%	236,210	139,964	65,096	31,813	12,378	6,120
**Haplotypes**	Marker #	690,056	412,396	194,629	92,937	36,882	18,370
	MAF>5%	159,255	92,061	43,491	21,642	8,605	4,294

Supplemental Table 2. Genetic variation detected among the maize groups with 867 maize inbred lines

Maize groups	Sample size	mSNP	SNP# MAF>5%	Haplotype (realized)	SNP# /mSNP	Haplptype /mSNP	PIC-SNP	PIC-Haplotype
Temperate	325	38,022	234,391	333,587	6.62	8.77	0.272	0.610
Iodent	32	37,708	200,244	158,093	6.64	4.19	0.223	0.486
Lancaster	64	37,977	209,039	200,474	6.62	5.28	0.228	0.501
LRC	37	37,922	221,404	192,361	6.63	5.07	0.245	0.554
PA	40	37,653	179,221	159,995	6.65	4.25	0.203	0.436
PB	36	37,785	200,445	181,408	6.64	4.80	0.221	0.500
Reid	60	37,828	182,734	176,127	6.63	4.66	0.207	0.444
SPT	56	37,957	217,471	204,281	6.62	5.38	0.236	0.535
Trop	288	38,019	216,176	402,015	6.62	10.57	0.253	0.605
Sweet	246	37,934	194,115	453,978	6.62	11.97	0.226	0.582

Heterotic groups in temperate maize: Iodent, Lancaster, LRC, PA, PB, Reid and SPT

Supplemental Table 3. 40K mSNPs, 251 SNPs and 159K haplotypes identified in different genomic regions using 867 maize inbred lines (CDS = exon)

Marker types		UTR5	Intergenic	CDS	UTR3	Intronic	Total
40K high-PIC	#	644	28544	1420	1477	5949	38034
SNPs	%	1.69	75.05	3.73	3.88	15.64	
251K SNPs	#	3585	194198	7794	8930	37124	251630
	%	1.42	77.18	3.10	3.55	14.75	
159K Haplotypes	#	3202	114155	8568	7245	26085	159255
	%	2.01	71.68	5.38	4.55	16.38	

Supplemental Table 4. Distribution of numbers and frequencies of insertion and deletion identified with 251K SNPs in genic and intergenic regions

	UTR5	Intergenic	CDS	UTR3	Intronic	Total
Insertion	40 (1.12)	1678 (0.86)	35 (0.45)	87 (0.97)	327 (0.88)	2167
Deletion	53 (1.48)	2893 (1.49)	34 (0.44)	133 (1.49)	520 (1.40)	3633
Total	93 (2.59)	4571 (2.35)	69 (0.89)	220 (2.46)	847 (2.28)	5800

The numbers in the parentheses are frequencies (%), calculated by comparing the number of insertion or deletion with the total number of SNPs identified in the same genomic region as shown in Supplemental Table 3.

Supplemental Table 5. LD decay distances estimated by different types of markers from genic and intergenic regions with different germplasm groups

**Germplasm**	**Marker types**	**Genomic regions**	**Decay distance (Kb)**	
			**r^2=0.1**	**r^2=0.2**
All germplasm	251K SNPs	Genic	12	2
		Intergenic	73	5
		Total	40	3
	40K high-PIC SNPs	Genic	26	9
		Intergenic	149	39
		Total	114	31
Sweet	251K SNPs	Genic	33	5
		Intergenic	195	7
		Total	180	5
	40K high-PIC SNPs	Genic	110	18
		Intergenic	499	95
		Total	480	79
Temperate	251K SNPs	Genic	29	4
		Intergenic	170	6
		Total	129	4
	40K high-PIC SNPs	Genic	38	13
		Intergenic	295	88
		Total	306	57
Tropical	251K SNPs	Genic	7	2
		Intergenic	38	3
		Total	26	3
	40K high-PIC SNPs	Genic	21	5
		Intergenic	89	36
		Total	80	7

Supplemental Figures

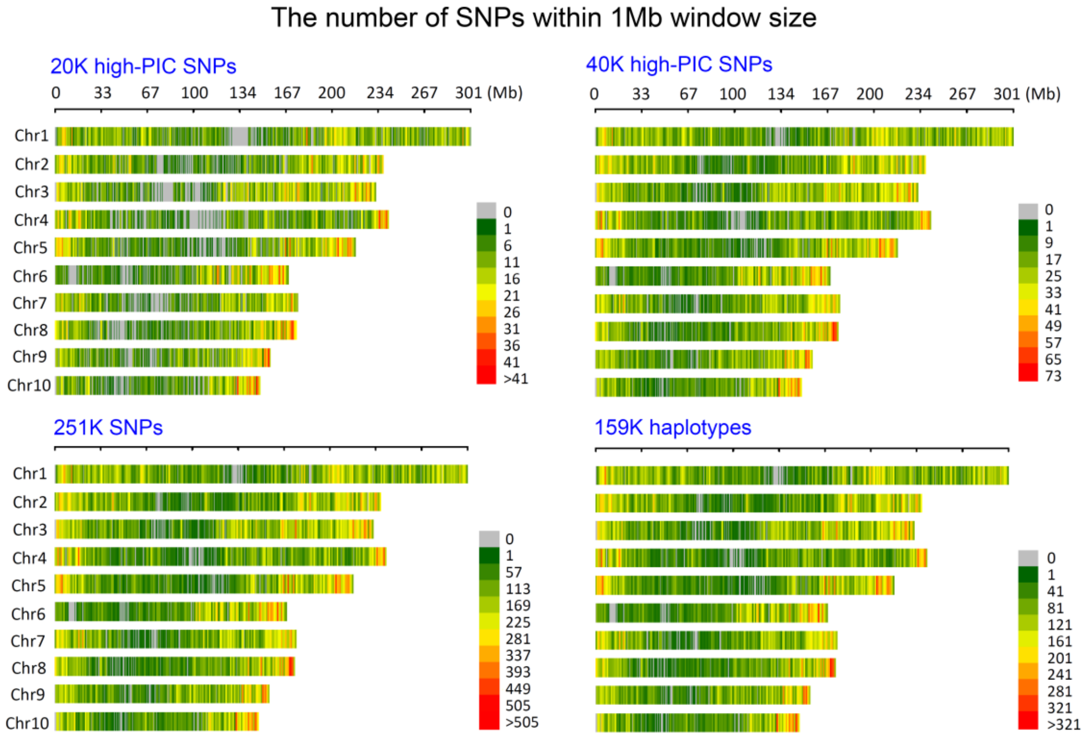

Supplemental Figure 1. Distribution of GBTS markers on maize chromosomes. Chromosome distribution was provided for 20K high-PIC SNPs, 40K high-PIC SNPs, 251K SNPs and 159K haplotypes. Marker density is indicated by bar color, and each bar represents 1-Mb window.

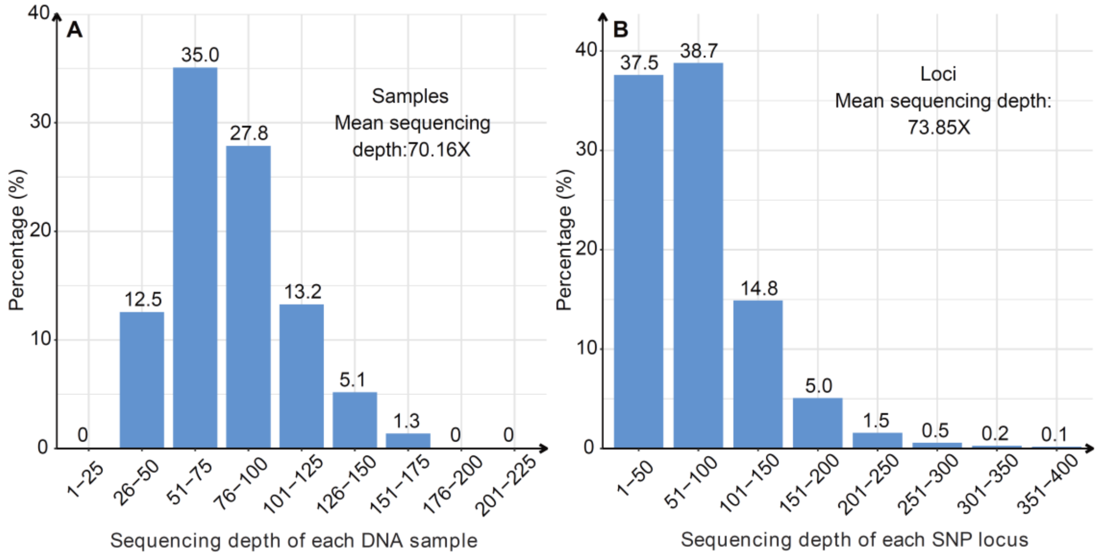

Supplemental Figure 2. Sequencing depths for genotyping. Distribution of sequencing depths for samples (867 maize inbred lines; A) and loci (40K mSNP loci; B).

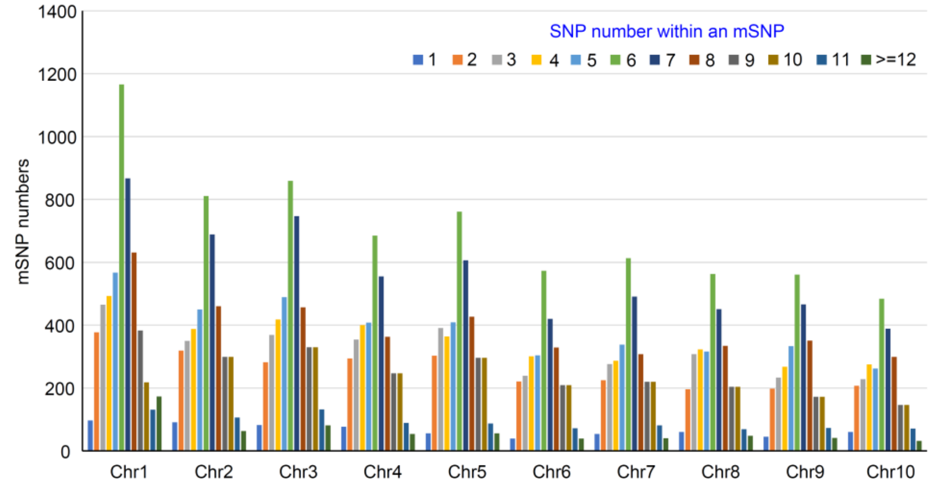

Supplemental Figure 3. Frequency distribution of 40K mSNPs on chromosomes. The numbers of the SNPs covered by mSNPs are coded by different colors.

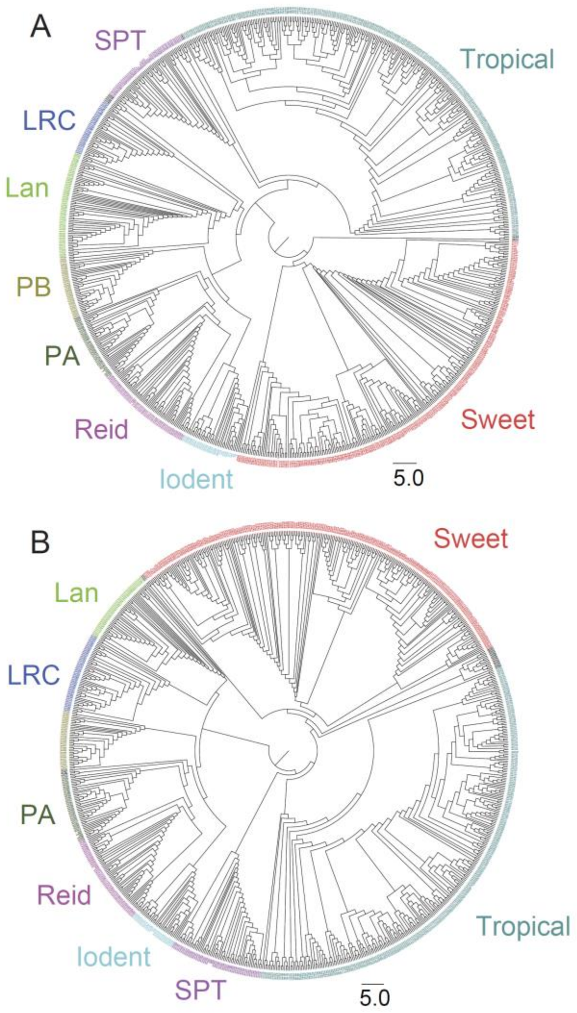

Supplemental Figure 4. Evaluation of markers by phylogenetic analysis. Phylogenetic trees constructed using maize inbred lines for the germplasm sample combined (n=867) using UPGMA and two marker types, 40K high-PIC SNPs (A) and 251K SNPs (B). Identical groups in the two trees can be identified by flipping the relevant tree branches.

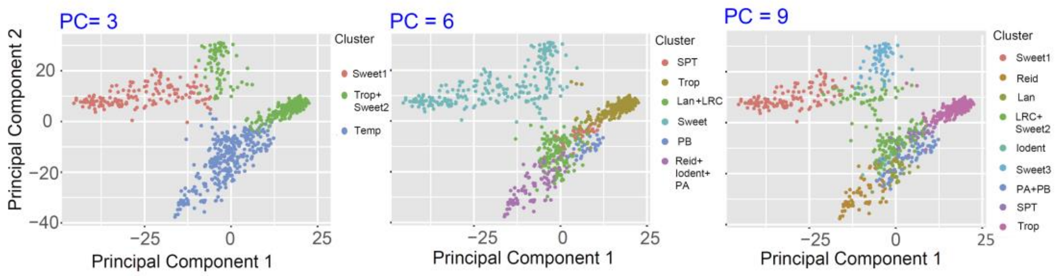

Supplemental Figure 5. Evaluation of markers by principal component analysis. Pairwise Modified Rogers' distances were estimated for 867 maize inbred lines using 40K high-PIC SNPs.

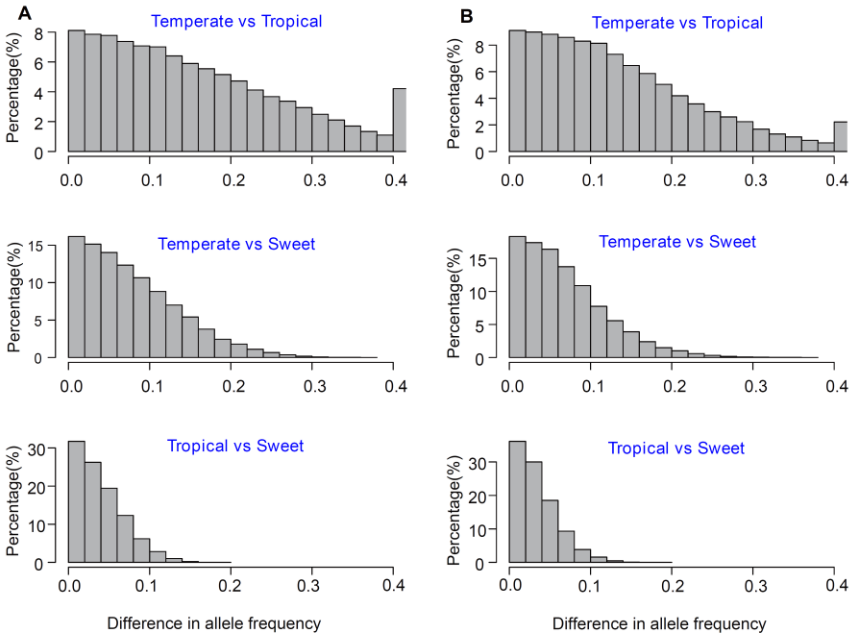

Supplemental Figure 6. Differentiation of allele frequencies between maize groups. The groups of 867 maize germplasm accessions were revealed by 40K high-PIC SNPs (A) and 251K SNPs (B). Comparisons between three germplasm groups: Temperate versus Tropical, Temperate versus Sweet, and Tropical versus Sweet.

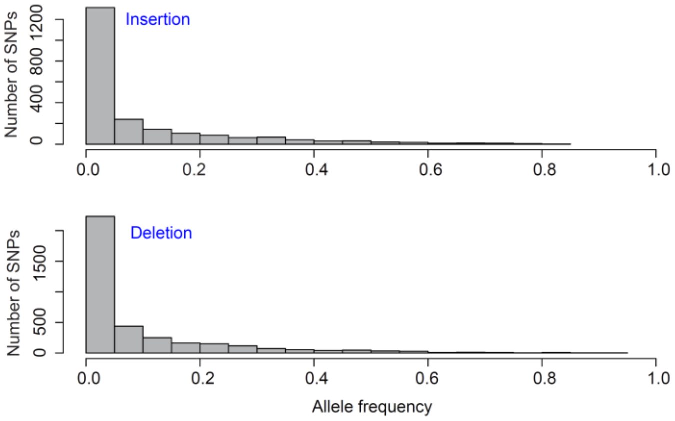

Supplemental Figure 7. Distribution of frequencies for insertions and deletions. A total of 251K SNPs were used for calculation of allele frequencies.
