# Supplementary material for: Development of high-resolution multiple-SNP arrays for genetic analyses and molecular breeding through genotyping by target sequencing and liquid chip
Source: Plant Commun. 2021 Aug 9;2(6):100230. doi: 10.1016/j.xplc.2021.100230 (PMC8577115; doi:10.1016/j.xplc.2021.100230)
Supplement: Document S2. Article plus supplemental information [file mmc2.pdf]

Plant Communications
Resource Article

CellPress Partner Journal

Development of high-resolution multiple-SNP arrays for genetic analyses and molecular breeding through genotyping by target sequencing and liquid chip

Zifeng Guo[1,9], Quannv Yang[2,9], Feifei Huang[3], Hongjian Zheng[4], Zhiqin Sang[5], Yanfen Xu[3], Cong Zhang[3], Kunsheng Wu[3], Jiajun Tao[3], Boddupalli M. Prasanna[7], Michael S. Olsen[7], Yunbo Wang[2,*], Jianan Zhang[3,8,*] and Yunbi Xu[1,2,4,6,*]

[1]Institute of Crop Science, Chinese Academy of Agricultural Sciences, Beijing 100081, China

[2]School of Food Science and Engineering, Foshan University/CIMMYT-China Tropical Maize Research Center, Foshan 528225, Guangdong, China

[3]MolBreeding Biotechnology Co., Ltd., Shijiazhuang 050035, China

[4]Crop Breeding and Cultivation Research Institute, Shanghai Academy of Agricultural Sciences/CIMMYT-China Specialty Maize Research Center, Shanghai 201403, China

[5]Xinjiang Academy of Agricultural Reclamation, Shihezi 832000, Xinjiang, China

[6]International Maize and Wheat Improvement Center (CIMMYT), El Batan Texcoco 56130, Mexico

[7]CIMMYT (International Maize and Wheat Improvement Center), ICRAF Campus, United Nations Avenue, Nairobi, Kenya

[8]National Foxtail Millet Improvement Center, Minor Cereal Crops Laboratory of Hebei Province, Institute of Millet Crops, Hebei Academy of Agriculture and Forestry Sciences, Shijiazhuang 050035, China

[9]These authors contributed equally to this article.

***Correspondence: Yunbo Wang (wyb0203@163.com), Jianan Zhang (algol@molbreeding.com), Yunbi Xu (y.xu@cgiar.org)**

https://doi.org/10.1016/j.xplc.2021.100230

ABSTRACT

Genotyping platforms, as critical supports for genomics, genetics, and molecular breeding, have been well implemented at national institutions/universities in developed countries and multinational seed companies that possess high-throughput, automatic, large-scale, and shared facilities. In this study, we integrated an improved genotyping by target sequencing (GBTS) system with capture-in-solution (liquid chip) technology to develop a multiple single-nucleotide polymorphism (mSNP) approach in which mSNPs can be captured from a single amplicon. From one 40K maize mSNP panel, we developed three types of markers (40K mSNPs, 251K SNPs, and 690K haplotypes), and generated multiple panels with various marker densities (1K–40K mSNPs) by sequencing at different depths. Comparative genetic diversity analysis was performed with genic versus intergenic markers and di-allelic SNPs versus non-typical SNPs. Compared with the one-amplicon-one-SNP system, mSNPs and within-mSNP haplotypes are more powerful for genetic diversity detection, linkage disequilibrium decay analysis, and genome-wide association studies. The technologies, protocols, and application scenarios developed for maize in this study will serve as a model for the development of mSNP arrays and highly efficient GBTS systems in animals, plants, and microorganisms.

Key words: multiple single-nucleotide polymorphisms, mSNPs, genotyping by target sequencing, GBTS, multiplexing PCR, sequence capture in-solution (liquid chip), linkage disequilibrium, LD

Guo Z., Yang Q., Huang F., Zheng H., Sang Z., Xu Y., Zhang C., Wu K., Tao J., Prasanna B.M., Olsen M.S., Wang Y., Zhang J., and Xu Y. (2021). Development of high-resolution multiple-SNP arrays for genetic analyses and molecular breeding through genotyping by target sequencing and liquid chip. Plant Comm. **2**, 100230.

Published by the Plant Communications Shanghai Editorial Office in association with Cell Press, an imprint of Elsevier Inc., on behalf of CSPB and CEMPS, CAS.

This is an open access article under the CC BY license (http://creativecommons.org/licenses/by/4.0/).

INTRODUCTION

Assisted by DNA markers and other genomics tools, genetic research and breeding have been accelerated during the past two decades, particularly in government-funded breeding programs and multinational breeding companies, where established breeding facilities and platforms can be shared across countries and crop plants (Crosbie et al., 2006; Bernardo, 2008; Collard and Mackill, 2008; Xu et al., 2017b; Voss-Fels et al., 2019; Watt et al., 2020). However, such efforts have been largely constrained in developing countries and small- and medium-size companies by a lack of high-throughput and cost-effective genotyping platforms due to limited funds, resources, and platforms that can be shared across the community (Kuchel et al., 2005; Collard and Mackill, 2008; Xu and Crouch, 2008). Therefore, the development of cost-effective, flexible, user-friendly, and less-demanding platforms is crucial for large-scale commercial breeding in both industry and developing countries, and the current availability of genotyping platforms is one of the key limiting factors that must be improved and upgraded significantly.

As a major molecular tool, marker-assisted selection (MAS) has been widely applied through the genotyping of breeding materials to obtain desired targets with specific marker alleles and their combinations. Various types of DNA markers and genotyping platforms, from first-generation agarose gel-based genotyping of RFLP markers to sequencing- and chip-based genotyping of SNP markers, have been developed and used in MAS (Tanksley and Rick, 1980; Beckmann and Soller, 1986; Tanksley et al., 1989; Edwards and Johnson, 1994; Fan et al., 2003; Xu, 2010; Yan et al., 2010; Ganal et al., 2011; Xu et al., 2013; Unterseer et al., 2014; Rasheed et al., 2017; Sun et al., 2020). In addition to flexibility, lower costs, and reduced demands, full-genome coverage of DNA markers and high-throughput, automated genotyping platforms are two important requirements for many genetics and breeding application scenarios, including the development of functional DNA markers, genomic selection/prediction of complex traits, genome-wide fingerprinting of DNA variation, plant variety protection, and quality control.

To develop high-throughput molecular markers that cover whole genomes, chip- and sequencing-based technologies have been developed. In maize, several chip-based genotyping platforms have been established using Illumina and Affymetrix systems; these platforms contain from 1536 (Yan et al., 2010) to 50K–55K (TraitGenetics INRA and Syngenta) (Xu et al., 2017a) and 600K (Unterseer et al., 2014) SNP markers. Although chip-based genotyping is highly stable and reliable, it is dependent on commercial chip products with fixed markers and specific genotyping platforms.

Three sequencing-based strategies have been adopted to date. Full genome sequencing, as a complete solution for identifying all sequence variability, is still too expensive for genotyping many individuals, as required in genomics, genetics, and breeding. Partial or selective sequencing using restriction enzyme–digested DNA, generally called genotyping by sequencing (GBS) (Baird et al., 2008; Davey et al., 2011; Elshire et al., 2011; Bradbury, 2013; Huang et al., 2014), can be used to generate DNA markers to cover selected genomic regions. However, high-density SNP genotyping by this strategy must be backed up by optimized genotyping pipelines, with information available for a large number of genotyped samples and strong informatics support, to impute marker genotypes for some samples and loci (Marchini and Howie, 2010; Glaubitz et al., 2014). The third sequencing-based strategy involves the capture of targeted genomic loci by probes. First developed in animals and more recently in plants, this genotyping by target sequencing (GBTS) strategy is known by different names (Tewhey et al., 2009; Mamanova et al., 2010; Yang et al., 2013; Samorodnitsky et al., 2015; Burridge et al., 2018; Johnson et al., 2018; Guo et al., 2019; Longeri et al., 2019; Bernardo et al., 2020; Zhang et al., 2020). GBTS can be performed for a small number of markers (several to 5K) through multiplexing PCR (GenoPlexs) (Zhang et al., 2020) and for a large number of markers (1K–20K) through capture-in-solution (liquid chip) with regular PCR plates (GenoBaits) (Guo et al., 2019). Genotyping can be performed using various currently available sequencing platforms. GBTS combines the advantages of solid chip-based technology (high stability and reliability) and GBS (high flexibility and cost-effectiveness). Its genotyping cost is significantly lower than that of chip-based genotyping when the same set of markers and samples are considered (Guo et al., 2019; Zhang et al., 2020). It generates sharable and accumulative marker data with less bioinformatics support. With the same marker panel (for example, 20K maize SNPs), multiple panels with 1K–20K SNPs can be generated by sequencing at different depths (Guo et al., 2019).

Although significant progress has been made recently in target sequencing and in-solution capture (Burridge et al., 2018; Johnson et al., 2018; Guo et al., 2019; Bernardo et al., 2020; Zhang et al., 2020), the current GBTS system still needs to be improved and optimized for DNA variation identification, cost reduction, and wide application in genomics, genetics, and molecular breeding. In this study, we first developed a new SNP array in maize that can be captured in solution, increasing the marker loci from 20K to 40K through optimized procedures. Second, a new protocol was developed to identify more than six SNPs from each individual amplicon; these were named multiple single-nucleotide polymorphisms (mSNPs). In this way, several times more SNPs (251K SNPs) can be generated from the same set of designed SNP assays for the same cost, further reducing the cost of genotyping. Third, after evaluation of the marker system and genotyping platform, a comparative analysis of three marker panels (40K mSNPs, 251K SNPs, and 159K haplotypes with minor allele frequencies greater than 5%) was performed to evaluate their power for DNA variation detection and genome-wide association study (GWAS). This improved GBTS system has great potential for development and implementation in all organisms, including plants, animals, and microorganisms.

RESULTS

Properties of mSNP markers: Basic statistics and marker diversity

A total of 83 916 target regions were selected as candidate mSNP loci and went through the alpha panel test using 96 temperate maize inbred lines, and 46 377 mSNP regions were retained. In the beta test, 647 regular maize inbreds were employed. The regions retained in the alpha test were ranked based on their average missing rates and average sequencing depths, and

Chr.	mSNP no.	SNP no.	SNP/mSNP	Haplotype no.			Haplotype no./mSNP			Amplicon length (bp)
				Theoretical	Realized	MAF > 5%	Theoretical	Realized	MAF > 5%	
1	5764	38 124	6.61	4 617 438	103 588	24 210	801.08	17.97	4.20	100.05
2	4440	29 804	6.71	5 756 514	84 033	18 915	1296.51	18.93	4.26	100.73
3	4695	31 757	6.76	6 096 148	87 670	19 808	1298.43	18.67	4.22	100.76
4	3898	25 245	6.48	4 041 218	65 938	15 945	1036.74	16.92	4.09	100.53
5	4128	27 099	6.56	5 177 220	72 799	17 101	1254.17	17.64	4.14	100.59
6	3054	20 244	6.63	4 804 682	55 570	12 948	1573.24	18.20	4.24	100.22
7	3218	21 356	6.64	4 752 174	59 578	13 580	1476.75	18.51	4.22	100.54
8	3158	20 469	6.48	3 584 280	57 419	13 035	1134.98	18.18	4.13	100.62
9	2975	19 649	6.60	3 844 442	55 030	12 420	1292.25	18.50	4.17	100.29
10	2704	17 883	6.61	2 693 956	48 431	11 293	996.29	17.91	4.18	100.24
Sum	38 034	251 630	6.62	45 368 072	690 056	159 255	1192.83	18.14	4.19	100.46

Table 1. Basic statistics for 40K mSNPs generated using GBTS and evaluated by 867 maize inbred lines.

6377 loci were removed. As a result, 40 000 mSNP markers were finally selected to cover the whole genome (supplemental Figure 1). The selection of mSNP loci was targeted to achieve high variability at each locus in order to enable the amplification of multiple SNPs from each amplicon. The actual number of mSNPs that can be achieved in a designed mSNP panel depends on the sequencing depth at which a given number of designed mSNPs can be covered. The panel of highly diverse maize germplasm used throughout this study consisted of 867 inbred lines from around the world, including three germplasm groups: temperate and tropical regular maize and sweet corn. A total of 40K mSNPs and 260K SNPs were identified, among which 38.0K mSNPs and 251K SNPs had a minor allele frequency (MAF) >5%, by sequencing at average depths of 70.16X for the samples and 73.85X for the markers (supplemental Figure 2). A high level of concordant genotype calls (97.8%–99.7%) was observed between two biological replicates for each of the 11 tested inbred lines. A total of 6.3 billion reads and 1880 Gb DNA were sequenced for the combined germplasm samples.

Each mSNP locus had an average of 6.62 SNPs with a range of 1–29, and 12 757 (34.7%) of the mSNP loci had 6 or 7 SNPs (Table 1 and supplemental Figure 3). From multiple SNPs identified from each mSNP (covering an average of 100.5 bp), the number of haplotypes could be inferred theoretically. Taking all mSNP loci together, 45M haplotypes could be inferred from the identified SNPs. However, only a portion of the theoretical haplotypes (690K, 1.52%) could be found or realized in our real dataset, with 159K haplotypes (0.34%) having MAF > 5%. Each mSNP had 1192.8 theoretical, 18.1 realized, and 4.2 highly frequent haplotypes, respectively. More mSNPs, SNPs, and haplotypes were identified on longer chromosomes, with the largest values for chromosome 1 and the smallest for chromosome 10.

In the following data analyses, the tested markers were classified into five categories: (1) 40K high-PIC SNPs (one SNP with the highest polymorphic information content (PIC) value from each mSNP), (2) 40K random SNPs (one SNP with an intermediate PIC value from each mSNP), (3) 251K SNPs (all the SNPs at 40K mSNP loci with MAF > 5%), (4) 690K haplotypes (realized), and (5) 159K haplotypes (with MAF > 5%).

Multiple marker panels developed from 40K mSNPs

We developed multiple mSNP panels from the same mother panel (40K mSNPs). Based on PIC values, the six developed mSNP panels each contained a subset of mSNPs (supplemental Table 1). The 20K mSNP panel was first selected based on PIC values (from high to low) and chromosomal distribution (supplemental Figure 1), and then the 10K, 5K, 2K, and 1K panels were selected successively. The 1K mSNPs had the highest average PIC value and were still evenly distributed across the genomic regions. The numbers of mSNPs included in the multiple panels were very close to the target numbers, as indicated by both high-PIC SNPs and random SNPs. The total SNPs and haplotypes in the multiple panels decreased proportionally as the target mSNP numbers decreased from 20K to 1K. For example, the 1K panel contained 1000 high-PIC mSNPs, 1000 random SNPs, 6493 SNPs, and 18 370 haplotypes (supplemental Table 1).

To understand how much sequencing depth is needed to cover the target mSNPs at a given missing rate, sequencing data were analyzed for each target mSNP number (Figure 1). In general, as sequencing quantity increased, the missing rate gradually decreased. For the same data quantity, the missing rate decreased with decreasing target marker numbers. Considering 40K mSNPs, each requiring a 300-bp read to capture the mSNP region, and a desired sequencing depth of 100X, the sequence quantity required for each sample is 40K × 300 bp ×100 = 1200 Mb. To control the missing rate below 5.0%, 1200 Mbp (100X) must be sequenced for the 40K mSNP panel, compared with 600 Mb (50X), 500 Mb (41X), 400 Mb (33X), 300 Mb (25X), and 300 Mb (25X) for the 20K, 10K, 5K, 2K, and 1K mSNP panels, respectively. When the number of target markers was reduced by half, the sequencing data could be reduced by 20%–50%. For each target marker set, highly consistent distribution of missing rates between the 40K mSNPs

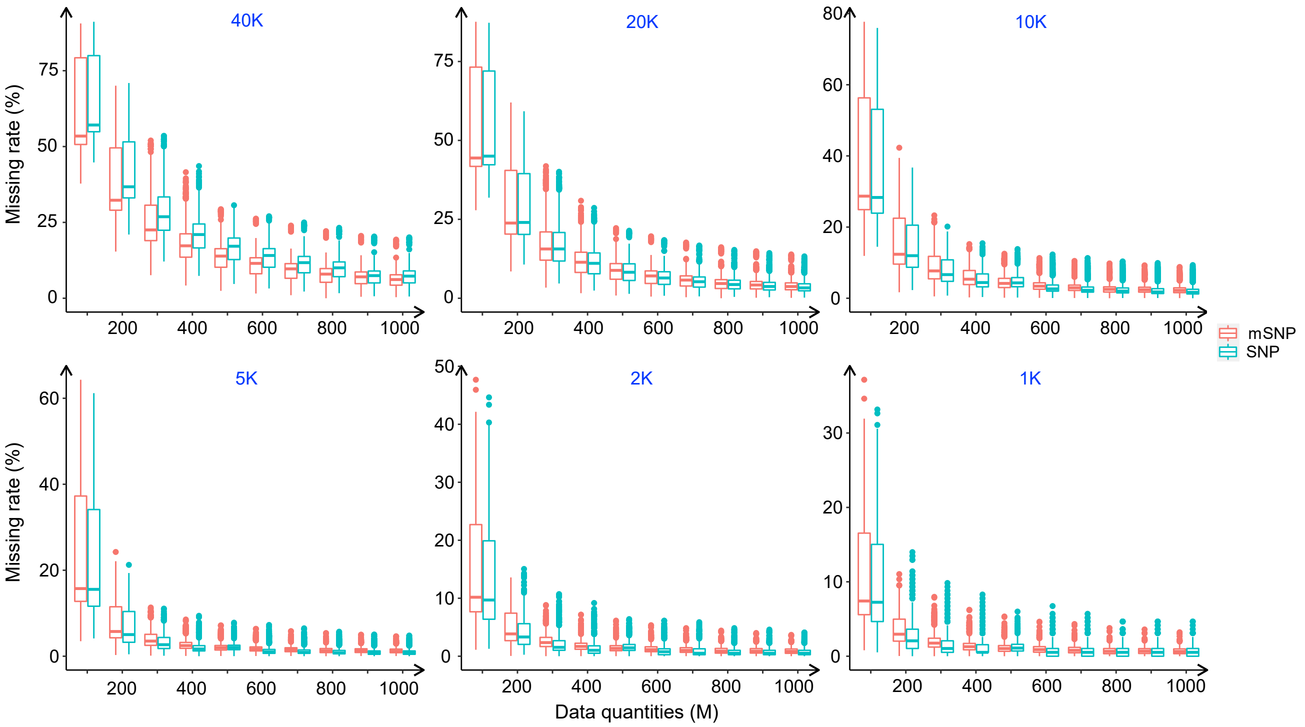

Figure 1. Genotyping data generated for multiple mSNP panels.
Missing rates and the sequencing quantities required to develop multiple mSNP panels with different numbers of target loci (1K to 40K) and their corresponding SNPs, calculated for both mSNP and SNP markers.

and the 251K SNPs was obtained. This is understandable, as once an mSNP locus has been covered by a read, its corresponding SNPs are also covered. Therefore, the sequencing depth required for the identification of all SNPs is almost the same as that required for their corresponding target mSNPs (Figure 1).

High-resolution classification of diverse germplasm

UPGMA (unweighted pair-group method with arithmetic means) trees were constructed with the 867 maize inbred lines using both 40K high-PIC SNPs and 251K SNPs. Almost identical phylogenetic relationships were identified with both marker panels, revealing nine groups, including the eight heterotic groups (Reid, SPT, LRC, Lancaster, PA, PB, Iodent, and Tropical) reported previously using 20K GBTS markers (Guo et al., 2019) and SNP chips (Remington et al., 2001; Lu et al., 2009; Xu et al., 2017a), as well as a new group of sweet maize (supplemental Figure 4). Some minor differences were found between the two trees, and higher resolution was obtained with the 251K SNPs when subgroups with closely related inbred lines were compared. Seven subgroups in the group "Temperate" were consistent with the heterotic groups established by pedigree information and breeders' experience with inbreds' combining ability.

Principal component analysis (PCA) showed a good agreement with cluster analyses (supplemental Figure 5). When the number of selected principal components (*K*) was 3, PCA revealed a clear separation of the three major germplasm groups: temperate, tropical, and sweet. Some sweet maize lines were clustered with the tropical group, whereas others were separate from both the temperate and tropical groups. When *K* = 6, sweet maize became a separate group. Several temperate heterotic groups, including SPT and PB, appeared, and three heterotic groups (Reid, Iodent, and PA) remained together at this level. When *K* = 9, six of the seven temperate heterotic groups could be distinguished, whereas the sweet inbreds were dispersed into two independent subgroups, one of which stayed with LRC.

To reveal genetic differences among different maize groups, we examined the differences in allele frequencies among three pairwise comparisons: temperate versus tropical lines, temperate versus sweet lines, and tropical versus sweet lines (supplemental Figure 6). Most of the SNPs had very small allele frequency differences between groups. Using the 40K high-PIC SNPs, the greatest allele frequency difference (0.159) was found between temperate and tropical lines with a range of 0–0.757, followed by the difference between temperate and sweet lines (0.079, 0–0.379) and the difference between tropical and sweet lines (0.040, 0–0.189). A very similar pattern of allele frequency differences was found when the 251K SNPs were used, and the average differences for the three comparisons were 0.138 (0–0.757), 0.091 (0–0.379), and 0.035 (0–0.189), respectively.

Comparison of genetic diversity in the 867 maize inbred lines revealed significant differences among groups (supplemental Table 2). Haplotypes showed much higher PIC values than SNPs, and temperate maize had the most realized haplotypes and the highest haplotype PIC among groups. Temperate maize had many more realized haplotypes and haplotypes per

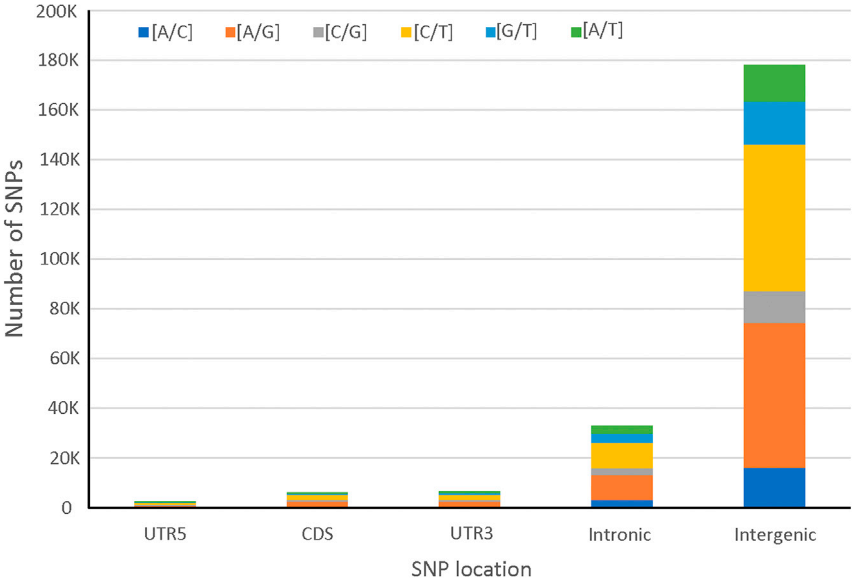

Figure 2. SNP markers developed from different genomic regions.
Numbers and types (transitions versus transversions) of the 251K SNPs occurring in genomic regions (CDS, intronic, intergenic, introns, and 5′ and 3′ UTRs).

mSNP, whereas the three maize groups did not differ significantly in hosted SNPs and SNPs per mSNP. The numbers of haplotypes and haplotype PIC values were much higher among heterotic groups (333 587 haplotypes with PIC of 0.610) than within heterotic groups (158 093–204 281 haplotypes with PIC values of 0.436–0.554). The heterotic group SPT had the highest haplotype number (204 281), whereas Iodent had the lowest (158 093). These results indicate that the development of heterotic groups in hybrid breeding has significantly reduced the genetic diversity within heterotic groups, and as a marker system, within-mSNP haplotypes had much greater power in genetic diversity analysis than single-SNP-based markers.

SNP markers in different genomic regions

To develop SNP markers to cover the whole genome, no intentional selection was made for specific nucleotides or genomic regions, and thus the markers included in the mother mSNP panel can be used to evaluate the specific genetic variation. The most frequent mSNP type was [C/T] (74 021, 32.74%), followed by [A/G] (32.09%), [G/T] (9.85%), [A/C] (9.25%), and [A/T] (8.52%). Most mSNPs (74.3%) were intergenic, 15.3% were intronic, and only 6.2% were from other regions (UTR3, UTR5, and CDS) (Figure 2). Intergenic regions contained 77.2% of the 251K SNPs and 71.7% of the haplotypes (supplemental Table 3).

To evaluate the power of markers in the detection of DNA variation, PIC and gene diversity (GD) were evaluated for markers from different genomic regions and for different marker types (Table 2). Compared with SNPs from other regions, CDS SNPs had relatively higher PIC and GD, as indicated by both the 40K high-PIC SNPs and random SNPs. Haplotypes in the UTR3 region had relatively higher PIC. When mSNPs were classified based on their SNP numbers, those containing fewer SNPs showed higher average estimates of PIC and GD. Relatively larger differences in PIC/GD among the mSNP types containing different SNPs were found in the UTR5 and CDS regions. The mSNP loci with three or fewer SNPs had a PIC of 0.298 and a GD of 0.296, compared with 0.272 (PIC) and 0.274 (GD) for the mSNP loci with eight or more SNPs.

Indels identified at SNP loci

Selection of high-quality target regions in a marker development procedure provides an opportunity to identify indels at each SNP locus; this has been largely neglected in most studies owing to mapping error from non-specific capture sequence or indels of a single base. In this study, indels were evaluated for their frequencies and distribution across the genome and in tested germplasm.

Across the 251K SNP loci, 2167 (0.86%) were insertions and 3633 (1.44%) deletions (supplemental Table 4 and supplemental Figure 7). Indels were present at very different frequencies, and most were very rare. On average, only 219.4 insertions and 336.8 deletions were observed across 251K SNPs in one inbred line. On the other hand, some indels were very frequent, and the highest frequencies for insertions and deletions were 83.6% and 91.2%, respectively. Indels were distributed throughout the maize genome, and telomeric regions contained many more than centromere regions, although they were not balanced between the two telomeres (Figure 3). In general, indels showed a very similar distribution pattern in the genome, and some chromosomal regions contained more variations than others. The most significant imbalance occurred on chromosome 6: most indels were located at the chromosomal end, and 72.2% of them were located in a small region on the second chromosomal arm (102–169.4M). Chromosomes 3, 5, and 8 were at the level of secondary imbalance. By comparing allele frequencies across genomic regions, insertions were found more frequently in UTR5 regions (1.12%, compared with 0.45%–0.97% for other regions) (supplemental Table 4).

Selection of high-PIC SNPs from each mSNP locus resulted in more SNPs with higher MAF, PIC, and GD in the 40K high-PIC SNPs than in the 251K SNPs, whereas the distribution of observed heterozygosity was almost the same between the two datasets (Figure 4). For example, 48.4% of the 40K high-PIC SNPs but only 14.8% of the 251K SNPs showed $0.40 < MAF \leq 0.50$, whereas 65.8% and 66.9% of the 40K high-PIC SNPs showed $PIC > 0.35$ and $GD > 0.45$, respectively. Although the 40K high-PIC SNPs had higher PIC and GD, they did exclude some rare alleles. It is advantageous to retain rare alleles when GWAS and genetic diversity analysis are performed. Therefore,

	Marker types	UTR5	Intergenic	CDS	UTR3	Intronic	Combined
PIC	40K high-PIC SNPs	0.346	0.343	0.347	0.341	0.342	0.343
	40K random SNPs	0.295	0.291	0.301	0.295	0.295	0.292
	251K SNPs	0.281	0.276	0.280	0.277	0.279	0.277
	mSNP (SNP ≦ 3)	0.298	0.276	0.296	0.286	0.287	0.280
	mSNP (SNP = 4–5)	0.281	0.276	0.277	0.286	0.282	0.278
	mSNP (SNP = 6–7)	0.279	0.28	0.279	0.279	0.280	0.280
	mSNP (SNP ≧ 8)	0.272	0.274	0.274	0.266	0.274	0.274
	690K haplotypes	0.633	0.650	0.650	0.670	0.664	0.653
GD	40K high-PIC SNPs	0.451	0.445	0.451	0.441	0.444	0.445
	40K random SNPs	0.366	0.360	0.376	0.360	0.366	0.362
	251K SNPs	0.347	0.340	0.348	0.341	0.344	0.341
	mSNP (SNP ≦ 3)	0.374	0.342	0.373	0.356	0.357	0.348
	mSNP (SNP = 4–5)	0.346	0.340	0.342	0.355	0.350	0.343
	mSNP (SNP = 6–7)	0.344	0.345	0.345	0.343	0.345	0.345
	mSNP (SNP ≧ 8)	0.335	0.336	0.337	0.325	0.336	0.336
	690K haplotypes	0.674	0.687	0.689	0.706	0.701	0.690

Table 2. PIC and GD for 40K high-PIC SNPs, 40K random SNPs, 251K SNPs, and 690K haplotypes covered by a 40K mSNP mother panel in intergenic and genic regions, evaluated by 867 maize inbred lines.

which of these two datasets should be used depends on the research purposes.

Linkage disequilibrium decay was well characterized by marker panels

To evaluate the power of the developed markers in genetic studies, linkage disequilibrium (LD) analysis was performed by marker type and with markers from different genomic regions for various germplasm groups (Figure 5 and supplemental Table 5). As indicated by LD analysis using the combined sample (867 inbred lines), the LD decay distance was much greater in the intergenic region than in the genic region. For r^2 = 0.1, the distances in intergenic versus genic regions estimated from the 40K high-PIC SNPs and 251K SNPs were 73K versus 12K and 149K versus 26K, respectively (Figure 5A). The same tendency was also observed across germplasm groups. When the germplasm sample combined with all genomic regions was considered, a much shorter LD decay distance (40K) was revealed with the 251K SNPs than with the 40K high-PIC SNPs (114K) (supplemental Table 5). Among the three maize groups, sweet maize decayed much more slowly, whereas tropical maize decayed much more rapidly (Figures 5B and 5C). The LD decay distances estimated with the 40K high-PIC SNPs for sweet, temperate, and tropical maize groups were 480K, 306K, and 80K, respectively, compared with 180K, 129K, and 26K estimated with the 251K SNPs. At the r^2 = 0.2 level, LD decay distances were much smaller (supplemental Table 5).

Our results largely confirm previous reports on maize LD decay (Remington et al., 2001; Ching et al., 2002; Palaisa et al., 2003; Tian et al., 2009; Yan et al., 2009; Chia et al., 2012) and also provide more details on different maize groups and the power of different marker types and markers from different genomic regions. The 251K SNPs, created by the development of multiple SNPs from each amplicon, reduced the LD decay distance by half compared with the regular marker type with only one SNP per amplicon. These results also indicate that tropical maize is more diverse and contains more rare alleles than temperate and sweet maize, and genic markers and the 251K SNPs are more powerful in LD analysis than their counterparts (intergenic markers and the 40K high-PIC SNPs).

High-resolution GWAS for cob color

To evaluate the power and accuracy of different marker types in GWAS, a major gene-controlled, highly heritable trait, cob color, was selected as an example. With the most stringent threshold of –log (0.01/the number of hypotheses tested), 20, 72, 129, and 72 SNPs on chromosome 1 were found to be significantly associated with cob color using four marker types: 40K random SNPs, 40K high-PIC SNPs, 251K SNPs, and 690K haplotypes, respectively (Figure 6). Using these marker types, 11, 19, 69, and 47 SNPs were identified in a 1.0-Mb region surrounding the most significant SNPs. The highest $-\log_{10} P$ value of 54.5 was obtained with the 251K SNPs and was much higher than the threshold and than that reported in a previous association study (Xie et al., 2013). The phenotypic variation explained by the most significant SNP was 9.0% for the 40K random SNPs, 6.8% for the 40K high-PIC SNPs, 34.8% for the 251K SNPs, and 42.6% for the 690K haplotypes, indicating that mapping power was significantly increased by using mSNP markers and within-mSNP haplotypes. With the 251K SNPs, there were four SNPs each explaining >10.0% of the phenotypic variance, whereas with the 690K haplotypes, the SNP number was 18, indicating that within-mSNP haplotypes improved the GWAS with the combined signal from multiple SNPs. The gene *pericarp color 1* (*P*1), which spans chromosome 1 from 48118782 to 48147883 and regulates red pigmentation in the cob, pericarp, and tassel glumes (Zhang and Peterson, 2005; Sekhon et al., 2007; Sidorenko and Chandler, 2008), was detected with all marker types. Compared with the 40K random and 40K high-PIC

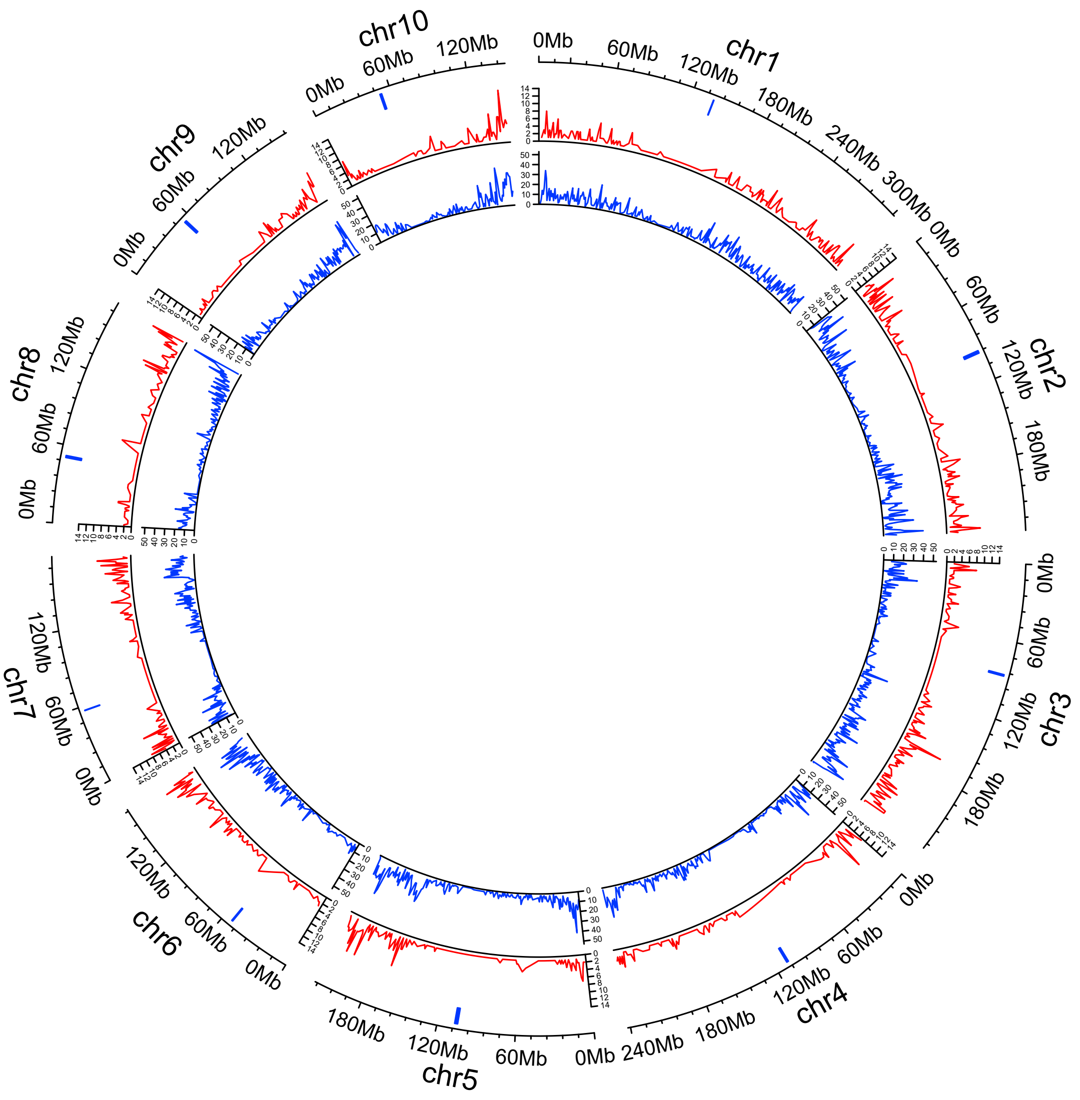

Figure 3. Distribution of frequencies for insertions and deletions.
The frequency was calculated for each 1-Mb interval across the maize genome. From outside to inside: chromosomes and their centromeres, insertion, deletion. Units on the circumference are megabases with centromeres indicated by blue bars.

SNPs, the most significant SNP (1_48204385) 57 Kb downstream of the *P*1 gene was identified with the 251K SNPs. Two SNPs, 1_48179494 and 1_48179550, from the same mSNP, were located 32 Kb upstream of the *P*1 gene, and significant SNPs were also detected with the haplotypes within this mSNP.

DISCUSSION

mSNPs serve as multi-purpose and powerful markers

Compared with genotyping technologies that detect only one SNP locus per amplification, mSNP markers, by which SNPs can be captured in solution (by liquid-phase chips), have contributed to the improvement of the genotyping platform in four ways. First, capturing multiple SNP markers at each target region increased the number of detectable SNPs 6.6-fold in this study. Second, 690K haplotypes, as an additional marker type, could be inferred from the multiple SNPs. Third, a core or high-PIC SNP marker set was developed from the SNPs with the largest PIC in each mSNP locus (amplicon). Fourth, detected DNA variation could be compared by within- and between-mSNPs and between SNPs and haplotypes. However, it should be noted that the multiple SNPs captured within an amplicon are more related to each other, although they are less likely to arise from alignment errors. First, to develop the mSNP array, target regions were selected from low-repeat genomic regions with high variability and more frequent SNPs. Second, regions with alignment errors were removed during the locus selection and optimization step. When a locus appears to be heterozygous in >50% inbred lines, an alignment error must have occurred. Therefore, the probability of mSNPs arising from alignment errors in this study should be very low, although it cannot be excluded.

mSNPs were developed to capture not only multiple SNPs (Table 1) but also different genomic regions (supplemental Figure 1). There are two important characteristics that enable mSNPs to serve as multi-purpose markers: the generation of multiple SNPs from one amplicon and the genotyping platform that

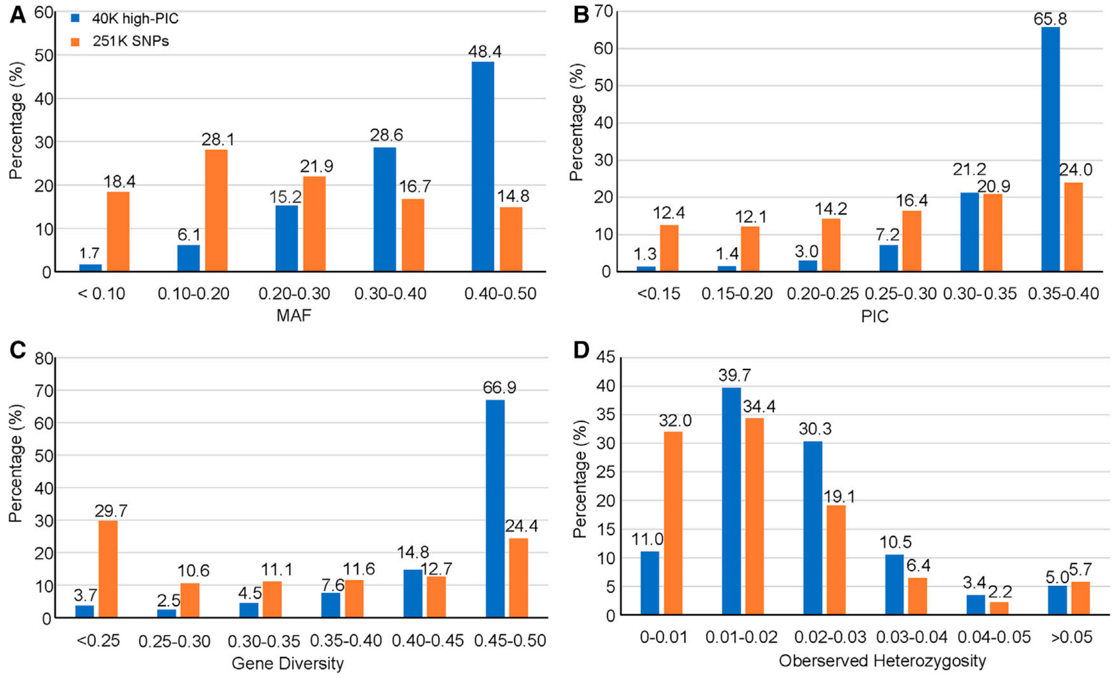

Figure 4. Marker characterization.
Minor allele frequency (MAF) **(A)**, polymorphic information content (PIC) **(B)**, gene diversity **(C)**, and observed heterozygosity **(D)** for 40K high-PIC SNPs and 251K SNPs as revealed in 867 maize inbred lines.

integrates highly efficient GBTS with capture-in-solution technology. Several properties of mSNPs contribute to their multipurpose applications. First, one universal marker system can be developed to target multiple marker types, including multiple and single SNPs, indels, and within-amplicon haplotypes. Second, these markers cover both intergenic and genic regions. Comparative analysis indicates that CDS SNPs have relatively higher PIC and GD estimates, and UTR3 haplotypes have relatively higher PIC estimates (Figure 2 and Table 2). Third, indels were systematically evaluated in this study and highly frequent indels identified. Telomeric regions contained many more indels than centromere regions (Figure 3). Fourth, the mSNPs can serve as a mother marker panel for the development of multiple SNP panels. One mother mSNP panel can be used to generate multiple marker panels with different numbers of mSNPs (from 1K to up to 40K) by sequencing at different depths (from 25X to 100X) (Figure 1). Traditionally, different marker sets must be developed to meet the requirements of specific application scenarios. With the GBTS marker system, multiple marker panels can be generated from one mother panel, as some genomic regions can be captured much more easily and can be covered by a smaller amount of sequence than others. The more easily the markers can be captured, the less sequencing depth will be required. As the capture capacity randomly varies across the genome, an even distribution of markers on the genome can be achieved for each target marker number, as shown in supplemental Figure 1. Therefore, users can select a specific marker number based on their own research objectives.

mSNPs are functionally suitable for multiple purposes. First, mSNPs can be developed for almost any organism, including plants, animals, and microorganisms. mSNPs are functionally suitable for all species, including those with polyploid chromosomes or very low genetic diversity. The numbers of SNPs identified in each amplicon range from 5.5 in wheat to 4.5 in rice and 6.6 SNPs in this study. Using the protocol developed here, high-density mSNP arrays have been developed in 13 plant and animal species to date (J.Z. et al., unpublished data). As an example of a polyploid species, wheat has been targeted for the development of mSNP markers. Because probes designed for target regions will capture all homologous fragments across the A, B, and D subgenomes, only homologous regions that are suitable for mSNPs were selected for probe design, bypassing regions that are less variable across the subgenomes. Thanks to the well-sequenced wheat subgenomes, 202 970 SNPs (5.1 SNPs/mSNP) were identified across 40 017 target regions (mSNPs) (J.Z. et al., unpublished data). Second, mSNP arrays are developed using variation information, and highly repetitive sequences are avoided; a perfect reference genome is therefore not required. Third, the marker number that can be included in an mSNP marker panel is very flexible, and it can be adapted to many potential application scenarios. The power of the 251K SNPs generated with the 40K mSNP array was not equivalent to that of the same number of SNPs obtained from separate amplicons, and thus the 40K mSNP array may not be powerful enough for performing high-resolution GWAS in highly diverse species such as maize. Application scenarios for the mSNP approach include DNA variation identification, germplasm fingerprinting, gene mapping and cloning, MAS, genomic prediction, and detection of transgenic events and genomic edits. In this study, a diverse maize germplasm collection of 867 inbred lines was evaluated, and detailed phylogenetic trees and high-resolution heterotic groups were constructed (supplemental

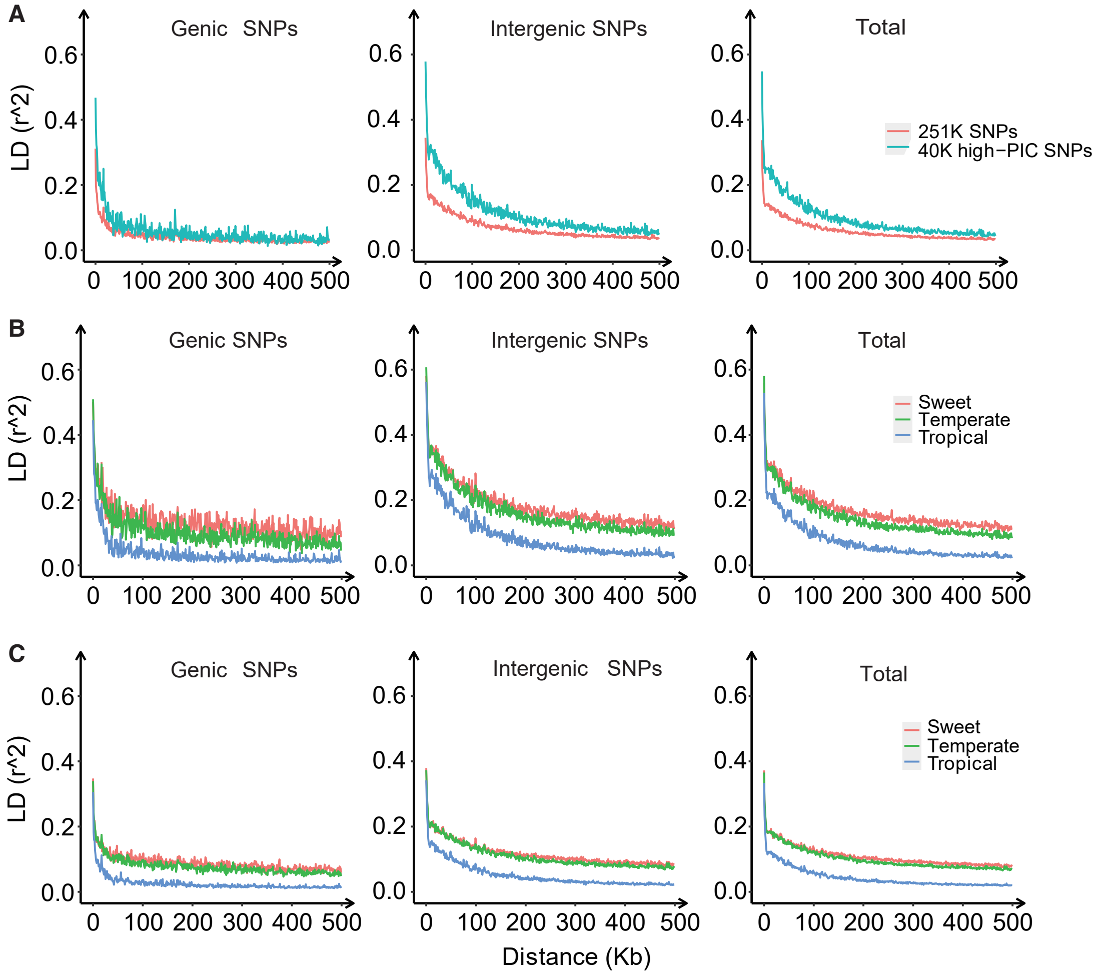

Figure 5. Evaluation of markers by linkage disequilibrium (LD) analysis.
Average LD decay by marker panels for the combined germplasm sample (n=867) **(A; top)**, maize germplasm groups (temperate, tropical, and sweet) (**B (middle)** for 40K high-PIC SNPs; **C (bottom)** for 251K SNPs), and also for markers from different genomic regions (intergenic SNPs, genic SNPs and total)

Figure 4). LD decay analysis indicated that much rapid decay could be revealed using more SNPs derived from the mSNPs (Figure 5). Using cob color as an example, GWAS with the 251K SNPs and haplotypes provided much higher resolution for gene mapping and candidate gene discovery compared with the 40K SNP-based analysis (Figure 6).

Advantages of mSNP approach when integrated with the GBTS platform

Traditional marker platforms such as TaqMan, KASP, or DNA arrays (chips) allow only one SNP per amplicon; otherwise, interference will occur. Thus, the potential of mSNP markers cannot be fully explored with such platforms. Integrated with the GBTS platform, the mSNP approach has several advantages for genotyping. First, the mSNP approach makes marker panel design more flexible and upgradable. Marker panels can be designed based on the requirements of very specific projects for any marker number and any sample size. Designed marker panels can then be upgraded by adding more markers to the existing marker panel when necessary. This kind of flexibility is not possible for chip-based markers, as, once designed, the marker number and sample size per chip are fixed.

The second advantage of the mSNP approach is associated with SNP calling and data management. Because SNP calls can be confirmed with each other using multiple SNPs within an mSNP locus, over 99.9% repeatability has been achieved regardless of the production batch, shelf-life, or laboratory associated with library construction and sequencing. Such levels of reliability and repeatability will greatly facilitate information management, including data collection, cross-comparison, accumulation, integration, and mining. Traditional GBS systems randomly sequence reduced genomes. The same set of samples, if genotyped at different times or across labs, will be sequenced at different genomic regions, making it difficult to compare and integrate data because of many missing calls. In some cases, missing alleles can be filled by imputation, if supported by high-quality reference genomes, fine-tuned haplotype maps, and a very large amount of accumulated genotypic data. Such resources exist in maize (Chia et al., 2012; Glaubitz et al., 2014; Bukowski et al., 2018) but are still not available for most plant or animal species. The mSNP approach

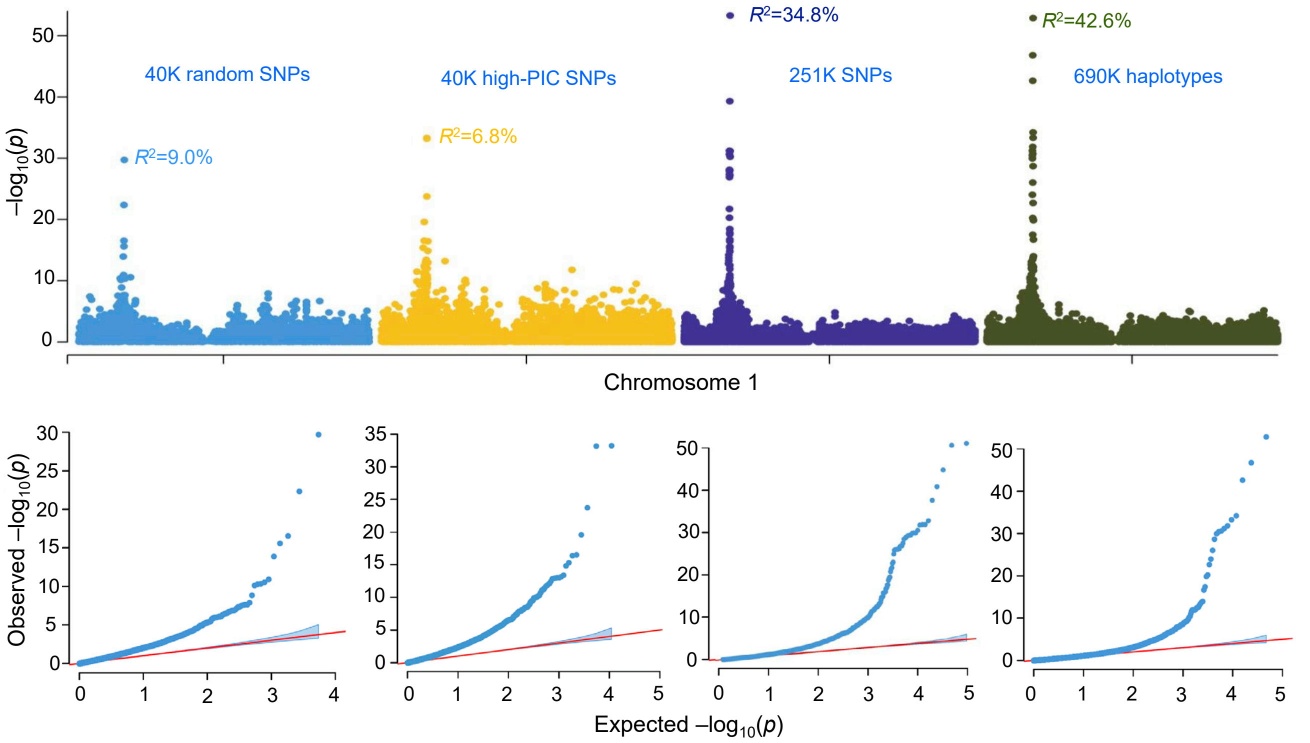

Figure 6. Evaluation of markers by genome-wide association study (GWAS).
Genome-wide association scans for cob color as an example to test the power of different marker types in gene mapping using 867 maize inbred lines. Manhattan plots (upper) and corresponding QQ plots (lower) are provided for 40K random SNPs, 40K high-PIC SNPs, 251K SNPs, and 690K haplotypes.

has no need for the filling of missing alleles during SNP calling because of the high-quality marker data and the fixed set of markers. Second, compared with traditional GBS based on a completely random fragment library, the mSNP approach was designed to capture 120-nt probes that have very good tolerance for variable sequences. Our data have shown that the designed probes have no bias but high sequencing depth (nearly 100X), even when base mutation (mismatch) has occurred in up to 10% of the bases in the probe (data not shown). The wide application of GBS at CIMMYT has revealed that traditional GBS has much higher genotyping error rates when heterozygous plants are genotyped (Wang et al., 2020). By contrast, one of our genetic mapping projects indicated that GBTS could successfully distinguish homozygotes from heterozygotes in a PHW43 × 8107 F2 population with 220 plants and 3544 polymorphic SNP markers, with an observed ratio close to 1:2:1 (24.6%:51.2%:24.2%) (Z.G. et al., unpublished data). Third, GBTS has been developed with no restrictions for public use, whereas traditional GBS has patents with a certain level of use restriction for research and commercial applications.

The mSNP system, when integrated with GBTS, has fewer demands in terms of genotyping platform, information management, and decision support. Compared with solid DNA chips that require specific genotyping facilities, mSNP markers can be genotyped by almost any currently available sequencing platform. A regular biology lab can establish a genotyping system for mSNPs without any additional technical support or professional bioinformatics assistance. mSNP information can be managed through a current lab information management system without any special improvement or upgrades because of the high-quality marker data and the same set of genotyped markers. The simplicity of data management in mSNP genotyping provides an opportunity to develop a smart model for SNP calling, data management, and reporting. In conclusion, the mSNP approach with improved GBTS developed in this study combines the advantages of both solid-chip and traditional GBS platforms; the former is more stable and reliable, whereas the latter is more flexible and cost-effective. The most significant limiting factor, however, is that the improved GBTS may not have a genotype-cost advantage at the level of super-high marker density.

The third advantage of the mSNP approach is that genotyping can be performed with two different systems, GenoPlexs based on multiplex PCR and GenoBaits based on capture-in-solution, covering a very flexible range of marker numbers from several markers to 40K mSNP loci or over 200K SNPs. For situations such as selection for single major genes, where only one or several markers will be genotyped, KASP would be the best choice at the current stage (Semagn et al., 2014; Thomson, 2014; Rasheed et al., 2016). An integrated mSNP and KASP system can meet all the requirements of application scenarios involving 1 to over 200K markers. On the other hand, KASP and other markers developed with known functions can be easily transformed into GBTS markers and included in an existing mSNP panel. For high-resolution GWAS, up to several million markers would be required for some plant species like maize that show high genetic diversity and very rapid LD decay, as revealed in this study (Figure 5 and supplemental Table 5). However, the marker number that can be covered by mSNPs in

this study will be sufficient for GWAS of most plants and animals (Figure 6).

The fourth advantage of the mSNP approach is its cost-effectiveness. Cost-effective genotyping platforms are a basic prerequisite for molecular breeding in small- and medium-size companies and for breeding programs in developing countries. GBTS has reduced the cost by at least half compared with a chip-based genotyping platform (Guo et al., 2019; Bernardo et al., 2020). The improved mSNP approach and GBTS system developed in this study will reduce costs by at least another half while increasing the number of SNP markers included in mSNP panels six-fold. For the 40K mSNP array developed in this study, the genotyping cost per sample is as low as \$14, including high-throughput DNA extraction (\$0.5), DNA library construction (\$2), probe synthesis (\$0.5), probe hybridization and wash (\$2), PCR (\$0.5), sequencing (\$8 for 1500 Mb in maize), and labor (including bioinformatics support) (\$0.5). The corresponding costs for the 20K, 10K, and 5K mSNP arrays developed through the GenoBaits protocol and the 2K and 1K mSNP arrays developed through GenoPlexs are as low as \$12, \$10, \$8.5, \$6, and \$5, respectively. In all cases, the genotyping costs are lower than the phenotyping costs for both complex traits (per inbred line, four rows) in Shunyi (\$25.18), Xinxiang (\$24.11), and Sanya (\$40.89) in China (Guo et al., 2019) and traits controlled by major genes (estimated as half the cost of complex traits). It should be noted that phenotyping costs in other countries may be significantly higher or lower than those in China. By genotyping a large batch of samples, the cost per sample can be further reduced. For example, when genotyping 100K samples through breeding initiatives, the cost per sample can be as low as \$10 and \$5 per sample for 10K and 1K mSNP arrays, respectively, because of significantly reduced labor costs and more efficient use of consumables. In addition, genotyping cost can be further reduced by imputation to minimize the sequencing depth, and this is particularly true for the high-PIC 40K SNPs. In this study, 155X coverage was required for low missing data with no imputation. An imputation approach will be incorporated into our genotyping platform and pipeline in the near future. Compared with non-GBS genotyping platforms, GBTS-based genotyping has much lower costs, except in cases with very few markers (1–5), such as genotyping by the KASP system, and super high-density chips with 200K or more SNPs. In conclusion, mSNP arrays are highly cost-effective, laying a strong foundation for the wide application of MAS in both multinational breeding companies and small-scale breeding programs in developing countries.

High repeatability and low genotyping cost make it possible to use mSNP markers to generate precise fingerprint profiles for almost all the final products developed in large-scale breeding programs. DNA variation, parental contribution, and selection profiles can be characterized and tracked based on genetic and chromosomal segments genotyped with high-density markers. Therefore, breeding initiatives or collaborative breeding programs for open-source breeding (http://gobiiproject.org) can be established among breeding companies and institutions to share genetic materials, breeding resources, benefits, and risks, as well as precision genotyping and phenotyping of all relevant breeding materials. The benefits produced in the breeding initiatives can be allocated among partners based on the genetic contributions of the parental lines involved. With the accumulation of very large genotypic, phenotypic, and envirotypic datasets and the optimization of genetic models and prediction methods, the genetic gain that can be achieved in breeding will be improved (Varshney et al., 2016). Through international or regional sharing platforms, open-source breeding may provide an opportunity for molecular breeding in resource-poor developing countries.

MATERIALS AND METHODS

Technical protocol for the development of mSNP markers

GBTS is based on target capture by complementary combination of the probe and the target sequence. First, probe sequences were designed according to target loci and synthesized by semiconductor-based in-situ synthesis with biotin modification. Second, probe hybridization formed double-stranded DNA with the target sequences from the constructed gDNA libraries. Third, streptavidin-coated magnetic beads were used to capture the biotin-modified probe, thereby capturing the target sequence. Finally, the captured target sequence was eluted, target amplified, and sequenced.

In traditional SNP genotyping, a pair of specific amplification primers (as in KASP and TaqMan) or a probe (for a chip) is designed based on each SNP marker, and only one SNP marker is generated per amplicon or hybridization. Therefore, the single identified SNPs form a uniform distribution on the genome. To maximize the use of the DNA sequences obtained from each amplicon, we developed a method for the identification of multiple SNPs in each single amplicon. The multiple SNPs developed from a single amplicon are called mSNPs. To develop the mSNP approach, we improved the currently available GBTS system (Guo et al., 2019) as follows (with more details in Figure 7):

1. The RNA probes used in hybridization were replaced by DNA probes, resulting in better uniformity and higher capture efficiency and experimental stability. Uniformity is defined as the proportion of the regions captured with 10% of the average depth required across the 40K loci to all regions. Capture efficiency, also called on-target rate, is defined as the proportion of useful data to all sequencing data.
2. The genomic regions with a Guanine-Cytidine (GC) content of 30%–70% were selected as candidate regions. Probes with a GC content of <30% are difficult to capture, reducing their capture ability, and genomic regions with a GC content above 70% have an adverse effect on PCR during sequencing.
3. The hybridization reagents and wash buffer were optimized, including the concentration of the saline-sodium citrate buffer. As a result, capture efficiency and uniformity were improved.
4. An improved library construction procedure free of quantification was developed, reducing the relevant cost by up to 50%.

The above optimization procedures greatly improved both uniformity and capture efficiency, two of the most important cost contributors; uniformity was as high as 95% and capture efficiency as high as 70% in the 40K mSNP array.

Development of mSNP marker panels in maize

To select SNP markers for the development of an mSNP panel, HapMap3 data (Bukowski et al., 2018) were used as a collection of SNP sources. First, we filtered the SNP loci in HapMap3 and selected 15M loci with MAF > 0.1 and missing rate <10%. Second, we used a 100-bp window to calculate the number of SNPs within each SNP locus, and the target fragments/loci prescreened in the first step, which were >2 and <15, were selected for subsequent analyses. Third, sequences located in repetitive regions were filtered out, and 662 534 target regions remained. Fourth, 62 504 target regions with PIC > 0.40 that were evenly distributed on chromosomes were retained. Finally, marker gaps >200 kb were imputed using the mSNPs that were filtered out in step 2 with SNP MAF > 0.1. Finally, 83 916 target regions were selected as candidate mSNP loci and used in the panel test.

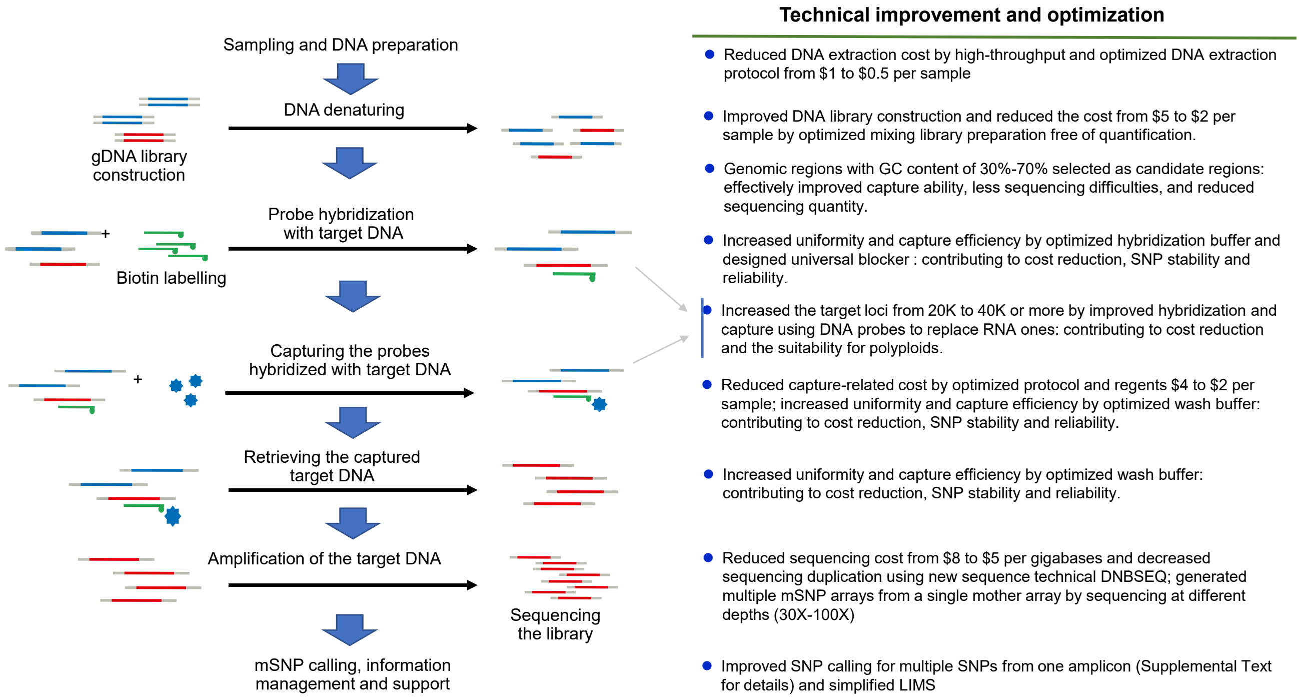

Figure 7. Technical procedure for improvement of genotyping by target sequencing (GBTS).
A chart for mSNP development through GBTS with highlighted technical improvements and optimization.

GenoBaits Probe Designer (Jianan Zhang, Molbreeding Biotech.) was used for probe design, and each locus was covered by two 110-nt probes. The probe set was synthesized by a semiconductor-based in-situ synthesis process. A 96-genotype panel (Guo et al., 2019) was used to evaluate these probes. When the capture uniformity (the ratio of the sequencing depth in the target region to the average sequencing depth of the sample) was <10%, the on-target rate (the ratio of reads covering the target region to all reads captured by the probe) was <50%, or the missing rate was <20%, the probes were removed from the probe set. As a result, 46 377 evenly distributed mSNP loci were selected for the beta test.

Using the 647 regular maize inbreds tested in this study as a genotype test panel, 46 377 SNP loci were ranked by their average missing rates and average sequencing depths, and 6377 loci were removed. Finally, 40 000 loci were selected to form a 40K mSNP marker panel. From this marker panel, we developed 20K, 10K, 5K, 2K, and 1K mSNP marker panels through the following steps. The genotype test panel was used to select 20K, 10K, 5K, 2K, and 1K mSNPs according to sequencing depth and even distribution on maize chromosomes. The higher the sequencing depth that can be captured at a marker locus, the less overall sequencing depth will be required in future genotyping; a lower minimum genotyping cost can therefore be achieved while keeping the marker missing score below the threshold. This means that the same set of 40K mSNPs can be used to generate 20K, 10K, 5K, 2K, and 1K mSNPs by sequencing at different depths.

Plant materials used in mSNP evaluation

To evaluate the marker panels developed in this study, we used 867 diverse maize germplasm accessions: 288 tropical/subtropical, 246 sweet maize, and 333 temperate inbred lines from China, the US, and the International Maize and Wheat Improvement Center (CIMMYT). One hundred forty-five of them were used previously in the HapMap3 project (Bukowski et al., 2018). Seventy-nine inbred lines from CIMMYT with CML numbers include maize germplasm adapted to diverse environments, including the Mexican lowland (CML161), Asian lowland (CML426), Mexican subtropics (CML312), and African mid-altitude region (CML206). The 246 sweet maize inbred lines were developed in the sweet maize breeding program of Foshan University by a pedigree method using hybrid germplasms introduced from temperate, subtropical, and tropical areas. Temperate inbreds have a good flavor and strong cold resistance; tropical inbreds have high yield and strong heat resistance; and subtropical inbreds are somewhere in between. All the sweet inbred lines contain one of two endosperm mutation gene combinations, *sh2* or *sh2su1*. Among the tested lines, 12 (B73, 78 010, LH61, PHP02, 465, 3189, D387, Chang7-2, Huangzaosi, TR0412, DTMA241, and VL0558) were genotyped twice as biological replicates.

DNA extraction, library construction, and probe hybridization

The genomic DNA was extracted from 15 pooled leaf samples using the high-throughput CTAB method (CAAS-CIMMYT Maize Molecular Breeding Laboratory methods, 2015). DNA quality and concentration were measured with a NanoDrop 2000 instrument. DNA libraries were constructed through DNA fragmentation, end-repair, adaptor ligation, and PCR. Subsequently, library hybridization capture was performed using the 40K mSNP panel developed above. All the other experimental steps followed those of Guo et al. (2019) with the modifications described above. The quality of the enriched libraries was assessed using an Agilent 2100 Bioanalyzer (Agilent Technologies, CA) and an Invitrogen Qubit 2.0 Fluorometer (Thermo Fisher Scientific, CA). Equivalent double-stranded DNA libraries were pooled and transformed into a single-stranded circular DNA library through DNA denaturation and circularization. DNA nanoballs were generated from single-stranded circular DNA by rolling circle amplification, quantified using a Qubit ssDNA Assay kit (Thermo Fisher Scientific, CA), loaded onto the flow cell, and sequenced with PE150 on the MGISEQ-2000 platform (MGI, Shenzhen, China).

In-silico analysis of sequence data

Raw sequencing reads were filtered to obtain clean reads. This was performed using fastp (version 0.20.0, -n 10 -q 20 -u 40) (Chen et al., 2018) to trim library adapters, remove reads with a low quality (phred score < 20) base ratio > 40%, and remove reads with >10 N bases. Clean reads

were aligned to the B73 reference genome using BWA software (Li and Durbin, 2009). Filtering of alignment results with mapping quality <30 was performed with Samtools (version 1.3) and the linux command awk. Sorting and reducing duplications were performed with Picard (version 2.1.1). Finally, variants were called with GATK (McKenna et al., 2010) (version v3.5-0-g36282e4, -dcov 1000000 -minIndelFrac 0.15 -glm BOTH -l INFO). mSNPs and haplotype variants were recorded and documented using Perl scripts written for this study (supplemental Note 1).

Marker data analysis and germplasm classification

Haplotypes were constructed when two or more SNPs were scored from a single amplicon. Because the individuals sampled came from inbred lines, the vast majority of SNP genotypes were homozygous, making haplotypes unambiguous. Theoretically, for mSNPs within a single amplicon, the number of haplotypes within the amplicon is 2^n, by which the number of theoretical haplotypes was determined.

Marker analysis was performed at three levels. At the level of marker types, four marker types were derived from the 40K mSNP mother panel: 40K high-PIC SNPs (SNPs with the highest PIC value from each mSNP), 40K random SNPs (SNPs with an intermediate PIC value from each mSNP), 251K SNPs (all the SNPs across 40K mSNP loci), and 159K haplotypes (MAF > 5%). At the level of genomic regions, SNP markers were classified into five categories: UTR5, intergenic, CDS, intronic, and UTR3. At the level of marker alleles, data analysis was performed for di-allelic SNPs and indels.

The missing rate, MAF, and heterozygosity were calculated for each SNP locus and haplotype. PIC, described by Botstein et al. (1980), was used to refer to the relative value of each marker with respect to the amount of polymorphism exhibited, which was estimated by

$$PIC = 1 - \left(\sum_{i=1}^{n} P_i^2\right) - \sum_{i=1}^{n-1}\sum_{j=i+1}^{n} 2P_i^2P_j^2$$

where P_i and P_j are the population frequencies of the ith and the jth alleles. GD is relevant to the sum of squares of allele frequencies and estimated as:

$$D = 1 - \sum_{i=1}^{n} P_i^2$$

where P_i is the frequency of the ith allele. The genetic distance between genotypes was evaluated using the average nucleotide difference of the genotype in TASSEL 5.0 (Bradbury et al., 2007). Genomic divergence between populations and pairwise nucleotide diversity within a population were calculated using the average value of all genotypes between populations and within populations. The maize germplasm groups were compared based on PIC, GD, and allele frequency difference.

Cluster analysis was performed using UPGMA, and groups were identified from the resulting phylogenetic tree. PCA was performed using TASSEL 5.0.

LD decay analysis and GWAS

LD decay between markers was quantified using the parameter r^2 (Hill and Robertson, 1968) estimated using Haploview software (Barrett et al., 2005) (version:4.2, -n -dprime -minGeno 0.5 -minMAF 0.01 -hwcutoff 0 -memory 60 000). The pairwise r^2 values were calculated for all SNPs in a 500-kb window. Then, average LD was calculated in increments of 1 kb according to marker distances. Finally, LD decay distances were profiled using the ggplot2 package in the R language.

To evaluate the power of different marker types in GWAS, data for cob color were collected for the tested maize inbreds. GWAS was performed with white cob coded as 0 and red as 1 using TASSEL 5.0 with MLM, taking into account both population structure and kinship matrix (***K***) between each pair of inbred lines. PCA was performed with TASSEL 5.0 to reduce false positives (Bradbury et al., 2007). Using the "no compression" and "population parameters, previously determined" (P3D) algorithms, an MLM was used to detect the marker-trait association. The MLM can be expressed as follows:

$$Y = X\beta + Zu + \varepsilon$$

where $\boldsymbol{Y}$ is the observed value vector, β is the fixed effect vector, which includes genetic markers and population structure factors, $\boldsymbol{u}$ is the random effect vector, $\boldsymbol{X}$ and $\boldsymbol{Z}$ are the known design matrices, and ε is the random residual effect vector. Manhattan plots were created in R software using the GWAS results.

SUPPLEMENTAL INFORMATION

Supplemental information is available at *Plant Communications Online*.

FUNDING

This research is supported by the National Key Research and Development Program of China (2016YFD0101803 and 2017YFD0101201), the Central Public-interest Scientific Institution Basal Research Fund (Y2020PT20), the Agricultural Science and Technology Innovation Program (ASTIP) of the Chinese Academy of Agricultural Sciences (CAAS) (CAAS-XTCX2016009), the Key Research Area and Development Program of Guangdong Province (2018B020202008), the Shijiazhuang Science and Technology Incubation Program (191540089A), and the Hebei Innovation Capability Enhancement Project (19962911D). Research activities at CIMMYT were supported by the Bill and Melinda Gates Foundation and the CGIAR Research Program MAIZE.

AUTHOR CONTRIBUTIONS

Yunbi X. and J.Z. conceived the project and designed the experiments. Yunbi X., J.Z., Y.W., M.S.O., and B.M.P. raised funding. Z.G., Q.Y., F.H., Yanfen X., C.Z., J.T., Q.Y., H.Z., J.Z., and Z.S. performed the experiments. Yunbi X., J.Z., Z.G., Q.Y., F.H., Yanfen X., C.Z., Y.W., K.W., and J.T. analyzed and interpreted the data. Yunbi X., J.Z., Z.G., F.H., and K.W. prepared and wrote the manuscript. All authors contributed to discussion of the manuscript.

ACKNOWLEDGMENTS

No conflict of interest declared.

Received: July 26, 2021
Revised: August 5, 2021
Accepted: August 6, 2021
Published: August 9, 2021

REFERENCES

Baird, N.A., Etter, P.D., Atwood, T.S., Currey, M.C., Shiver, A.L., Lewis, Z.A., Selker, E.U., Cresko, W.A., and Johnson, E.A. (2008). Rapid SNP discovery and genetic mapping using sequenced RAD markers. PLoS One **3**:e3376.

Barrett, J.C., Fry, B., Maller, J., and Daly, M.J. (2005). Haploview: analysis and visualization of LD and haplotype maps. Bioinformatics **21**:263–265.

Beckmann, J., and Soller, M. (1986). Restriction fragment length polymorphisms in plant genetic improvement. Oxford Surv. Plant Mol. Biol. Cell Biol. **3**:196–250.

Bernardo, A., Amand, P.S., Le, H.Q., Su, Z., and Bai, G. (2020). Multiplex restriction amplicon sequencing: a novel next-generation sequencing-based marker platform for high-throughput genotyping. Plant Biotechnol. J. **18**:254–265.

Bernardo, R. (2008). Molecular marker and selection for complex traits in plants: learning from the last 20 years. Crop Sci. **48**:1649–1664.

Botstein, D., White, R.L., Skolnick, M., and Davis, R.W. (1980). Construction of a genetic linkage map in man using restriction fragment length polymorphism. Am. J. Hum. Genet. **32**:314–331.

Bradbury, P. (2013). Applying Genotyping by Sequencing (GBS) to Corn Genetics and Breeding (Ithaca, NY: USDA-ARS/Cornell University).

Bradbury, P.J., Zhang, Z., Kroon, D.E., Casstevens, T.M., Ramdoss, Y., and Buckler, E.S. (2007). TASSEL: software for association mapping of complex traits in diverse samples. Bioinformatics **23**:2633–2635.

Bukowski, R., Guo, X., Lu, Y., Zou, C., He, B., Rong, Z., Wang, B., Xu, D., Yang, B., Xie, C., et al. (2018). Construction of the third generation Zea mays haplotype map. GigaScience **7**:1–12.

Burridge, A.J., Wilkinson, P.A., Winfield, M.O., Barker, G.L.A., Allen, A.M., Coghill, J.A., Waterfall, C., and Edwards, K.J. (2018). Conversion of array-based single nucleotide polymorphic markers for use in targeted genotyping by sequencing in hexaploid wheat (Triticum aestivum). Plant Biotechnol. J. **16**:867–876.

Chen, S., Zhou, Y., Chen, Y., and Gu, J. (2018). fastp: an ultra-fast all-in-one FASTQ preprocessor. Bioinformatics **34**:i884–i890.

Chia, J.M., Song, C., Bradbury, P.J., Costich, D., de Leon, N., Doebley, J., Elshire, R.J., Gaut, B., Geller, L., Glaubitz, J.C., et al. (2012). Maize HapMap2 identifies extant variation from a genome in flux. Nat. Genet. **44**:803–807.

Ching, A., Caldwell, K.S., Jung, M., Dolan, M., Smith, O.S., Tingey, S., Morgante, M., and Rafalski, A.J. (2002). SNP frequency, haplotype structure and linkage disequilibrium in elite maize inbred lines. BMC Genomics **3**:19.

Collard, B.C., and Mackill, D.J. (2008). Marker-assisted selection: an approach for precision plant breeding in the twenty-first century. Philos. Trans. R. Soc. Lond. Ser. B **363**:557–572.

Crosbie, T.M., Eathington, S.R., Johnson, G.R., Edwards, M., Reiter, R., Stark, S., Mohanty, R.G., Oyervides, M., Buehler, R.E., Walker, A.K., et al. (2006). Plant breeding: past, present and future. In Plant Breeding: The Arnel R. Hallauer International Symposium, K.R. Lamkey and M. Lee, eds. (Oxford, UK: Blackwell Press), pp. 3–50.

Davey, J.W., Hohenlohe, P.A., Etter, P.D., Boone, J.Q., Catchen, J.M., and Blaxter, M.L. (2011). Genome-wide genetic marker discovery and genotyping using next-generation sequencing. Nat. Rev. Genet. **12**:499–510.

Edwards, M., and Johnson, L. (1994). RFLPs for rapid recurrent selection. In Proceedings of Symposium on Analysis of Molecular Marker Data (Corvallis, Oregon: American Society of Horticultural Science and Crop Science Society of America Press), pp. 33–40.

Elshire, R.J., Glaubitz, J.C., Sun, Q., Poland, J.A., Kawamoto, K., Buckler, E.S., and Mitchell, S.E. (2011). A robust, simple genotyping-by-sequencing (GBS) approach for high diversity species. PLoS One **6**:e19379.

Fan, J.B., Oliphant, A., Shen, R., Kermani, B.G., Garcia, F., Gunderson, K.L., Hansen, M., Steemers, F., Butler, S.L., Deloukas, P., et al. (2003). Highly parallel SNP genotyping. Cold Spring Harb. Symp. Quant. Biol. **68**:69–78.

Ganal, M.W., Durstewitz, G., Polley, A., Bérard, A., Buckler, E.S., Charcosset, A., Clarke, J.D., et al. (2011). A large maize (*Zea mays* L.) SNP genotyping array: development and germplasm genotyping, and genetic mapping to compare with the B73 reference genome. PLoS One **6**:e28334.

Glaubitz, J.C., Casstevens, T.M., Lu, F., Harriman, J., Elshire, R.J., Sun, Q., and Buckler, E.S. (2014). TASSEL-GBS: a high capacity genotyping by sequencing analysis pipeline. PLoS One **9**:e90346.

Guo, Z., Wang, H., Tao, J., Ren, Y., Xu, C., Wu, K., Zou, C., Zhang, J., and Xu, Y. (2019). Development of multiple SNP marker panels affordable to breeders through genotyping by target sequencing (GBTS) in maize. Mol. Breed. **39**:37.

Hill, W., and Robertson, A. (1968). Linkage disequilibrium in finite populations. Theor. Appl. Genet. **38**:226–231.

Huang, Y.F., Poland, J.A., Wight, C.P., Jackson, E.W., and Tinker, N.A. (2014). Using genotyping-by-sequencing (GBS) for genomic discovery in cultivated oat. PLoS One **9**:e102448.

Johnson, M.G., Pokorny, L., Dodsworth, S., Botigué, L.R., Cowan, R.S., Devault, A., Eiserhardt, W.L., et al. (2018). A universal probe set for targeted sequencing of 353 nuclear genes from any flowering plant designed using k-medoids clustering. Syst. Biol. **68**:594–606.

Kuchel, H., Ye, G.Y., Fox, R., and Jefferies, S. (2005). Genetic and economic analysis of a targeted marker-assisted wheat breeding strategy. Mol. Breed. **16**:67.

Li, H., and Durbin, R. (2009). Fast and accurate short read alignment with Burrows-Wheeler transform. Bioinformatics **25**:1754–1760.

Longeri, M., Chiodi, A., Brilli, M., Piazza, A., Lyons, L.A., Sofronidis, G., Cozzi, M.C., et al. (2019). Targeted genotyping by sequencing: a new way to genome profile the cat. Anim. Genet. **50**:718–725.

Lu, Y., Yan, J., Guimarães, C.T., Taba, S., Hao, Z., Gao, S., Chen, S., Li, J., Zhang, S., Vivek, B.S., et al. (2009). Molecular characterization of global maize breeding germplasm based on genome-wide single nucleotide polymorphisms. Theor. Appl. Genet. **120**:93–115.

Mamanova, L., Coffey, A.J., Scott, C.E., Kozarewa, I., Turner, E.H., Kumar, A., Howard, E., Shendure, J., and Turner, D.J. (2010). Target-enrichment strategies for next-generation sequencing. Nat. Methods **7**:111–118.

Marchini, J., and Howie, B. (2010). Genotype imputation for genome-wide association studies. Nat. Rev. Genet. **11**:499–511.

McKenna, A., Hanna, M., Banks, E., Sivachenko, A., Cibulskis, K., Kernytsky, A., Garimella, K., et al. (2010). The Genome Analysis Toolkit: a MapReduce framework for analyzing next generation DNA sequencing data. Genome Res. **20**:1297–1303.

Palaisa, K.A., Morgante, M., Williams, M., and Rafalski, A. (2003). Contrasting effects of selection on sequence diversity and linkage disequilibrium at two phytoene synthase loci. Plant Cell **15**:1795–1806.

Rasheed, A., Hao, Y., Xia, X., Khan, A., Xu, Y., Varshney, R.K., and He, Z. (2017). Crop breeding chips and genotyping platforms: progress, challenges, and perspectives. Mol. Plant **10**:1047–1064.

Rasheed, A., Wen, W., Gao, F., Zhai, S., Jin, H., Liu, J., Guo, Q., Zhang, Y., Dreisigacker, S., Xia, X., et al. (2016). Development and validation of KASP assays for genes underpinning key economic traits in bread wheat. Theor. Appl. Genet. **129**:1843–1860.

Remington, D.L., Thornsberry, J.M., Matsuoka, Y., Wilson, L.M., Whitt, S.R., Doeblay, J., Kresovich, S., Goodman, M.M., and Buckler, E.S. (2001). Structure of linkage disequilibrium and phenotypic associations in the maize genome. Proc. Natl. Acad. Sci. USA **98**:11479–11484.

Samorodnitsky, E., Datta, J., Jewell, B.M., Hagopian, R., Miya, J., Wing, M.R., Damodaran, S., Lippus, J.M., Reeser, J.W., Bhatt, D., et al. (2015). Comparison of custom capture for targeted next-generation DNA sequencing. J. Mol. Diagn. **17**:64–75.

Sekhon, R.S., Peterson, T., and Chopra, S. (2007). Epigenetic modifications of distinct sequences of the p1 regulatory gene specify tissue-specific expression patterns in maize. Genetics **175**:1059–1070.

Semagn, K., Babu, R., Hearne, S., and Olsen, M. (2014). Single nucleotide polymorphism genotyping using Kompetitive Allele Specific PCR (KASP): overview of the technology and its application in crop improvement. Mol. Breed. **33**:1–14.

Sidorenko, L., and Chandler, V. (2008). RNA-dependent RNA polymerase is required for enhancer-mediated transcriptional silencing associated with paramutation at the maize p1 gene. Genetics **180**:183–1993.

Sun, C., Dong, Z., Zhao, L., Ren, Y., Zhang, N., and Chen, F. (2020). The Wheat 660K SNP array demonstrates great potential for marker-assisted selection in polyploidy wheat. Plant Biotechnol. J. **18**:1354–1360.

Tanksley, S.D., and Rick, C.M. (1980). Isozyme gene linkage map of the tomato: applications in genetics and breeding. Theor. Appl. Genet. **57**:161–170.

Tanksley, S.D., Young, N.D., Paterson, A.H., and Bonierbale, M.W. (1989). RFLP mapping in plant breeding: new tools for an old science. Bio/Technol. **7**:257–263.

Tewhey, R., Nakano, M., Wang, X., Pabón-Peña, C., Novak, B., Giuffre, A., Lin, E., Happe, S., Roberts, D.N., LeProust, E.M., et al. (2009). Enrichment of sequencing targets from the human genome by solution hybridization. Genome Biol **10**:R116.

Unterseer, S., Bauer, E., Haberer, G., Seidel, M., Knaak, C., Ouzunova, M., Meitinger, T., Strom, T.M., Fries, R., Pausch, H., et al. (2014). A powerful tool for genome analysis in maize: development and evaluation of the high density 600 k SNP genotyping array. BMC Genomics **15**:823.

Thomson, M.J. (2014). High-throughput SNP genotyping to accelerate crop improvement. Plant Breed. Biotechnol. **2**:195–212.

Tian, F., Stevens, N.M., and Buckler, E.S. (2009). Tracking footprints of maize domestication and evidence for a massive selective sweep on chromosome 10. Proc. Natl. Acad. Sci. U S A **106**:9979–9986.

Varshney, R.K., Singh, V.K., Hickey, J.M., Xu, X., Marshall, D.F., Wang, J., Edwards, D., and Ribaut, J.M. (2016). Analytical and decision support tools for genomics-assisted breeding. Trends Plant Sci **21**:354–363.

Voss-Fels, K.P., Cooper, M., and Hayes, B.J. (2019). Accelerating crop genetic gains with genomic selection. Theor. Appl. Genet. **132**:669–686.

Wang, N., Yuan, Y., Wang, H., Yu, D., Liu, Y., Zhang, A., Gowda, M., Nair, S.K., Hao, Z., Lu, Y., et al. (2020). Applications of genotyping-by-sequencing (GBS) in maize genetics and breeding. Sci. Rep. **10**:16308.

Watt, M., Fiorani, F., Usadel, B., Rascher, U., Muller, O., and Schurr, U. (2020). Phenotyping: new windows into the plant for breeders. Annu. Rev. Plant Biol. **71**:689–712.

Xie, C., Weng, J., Liu, W., Zou, Z., Hao, Z., Li, W., Li, M., Guo, X., Zhang, G., Xu, Y., et al. (2013). Zea mays (L.) P1 locus for cob glume color identified as a post-domestication selection target with an effect on temperate maize genomes. Crop J **1**:15–24.

Xu, Y., Xie, C., Wan, J., He, Z., and Prasanna, B.M. (2013). Marker-assisted selection in cereals: platforms, strategies and examples. In Cereal Genomics II, P.K. Gupta and R.K. Varshney, eds. (Dordrecht: Springer Science+ Business, Media), pp. 375–411.

Xu, C., Ren, Y., Jian, Y., Guo, Z., Zhang, Y., Xie, C., Fu, J., Wang, H., Wang, G., Xu, Y., et al. (2017a). Development of a maize 55K SNP array with improved genome coverage for molecular breeding. Mol. Breed. **37**:20.

Xu, Y., Li, P., Zou, C., Lu, Y., Xie, C., Zhang, X., Prasanna, B.M., and Olsen, M.S. (2017b). Enhancing genetic gain in the era of molecular breeding. J. Exp. Bot. **68**:2641–2666.

Xu, Y. (2010). Molecular Plant Breeding (Wallingford, UK: CABI Publishing).

Xu, Y., and Crouch, J.H. (2008). Marker-assisted selection in plant breeding: from publications to practice. Crop Sci. **48**:391–407.

Yan, J., Shah, T., Warburton, M.L., Buckler, E.S., McMullen, M.D., and Crouch, J. (2009). Genetic characterization and linkage disequilibrium estimation of a global maize collection using SNP markers. PLoS One **4**:e8451.

Yan, J., Yang, X., Shah, T., Sánchez, H., Li, J., Warburton, M., Zhou, Y., Crouch, J.H., and Xu, Y. (2010). High-throughput SNP genotyping with the GoldenGate assay in maize. Mol. Breed. **25**:441–451.

Yang, L., Yin, X., Wu, L., Chen, N., Zhang, H., Li, G., and Ma, Z. (2013). Targeted exome capture and sequencing identifies novel PRPF31 mutations in autosomal dominant retinitis pigmentosa in Chinese families. BMJ Open **3**:e004030.

Zhang, F., and Peterson, T. (2005). Comparisons of maize pericarp color 1 alleles reveal paralogous gene recombination and an organ-specific enhancer region. Plant Cell **17**:903–914.

Zhang, J., Yang, J., Zhang, L., Luo, J., Zhao, H., Zhang, J., and Wen, C. (2020). A new SNP genotyping technology Target SNP-seq and its application in genetic analysis of cucumber varieties. Sci. Rep. **10**:5623.

Plant Communications, Volume *2*

Supplemental information

Development of high-resolution multiple-SNP arrays for genetic analyses and molecular breeding through genotyping by target sequencing and liquid chip

Zifeng Guo, Quannv Yang, Feifei Huang, Hongjian Zheng, Zhiqin Sang, Yanfen Xu, Cong Zhang, Kunsheng Wu, Jiajun Tao, Boddupalli M. Prasanna, Michael S. Olsen, Yunbo Wang, Jianan Zhang, and Yunbi Xu

Supplemental Text. The script used for calling mSNPs from each amplicon

1．Quality Control

```
fastp -i 2019B97_1.fq.gz   -I 2019B97__2.fq.gz    -o 2019B97_clean_1.fq.gz -O
2019B97_clean_2.fq.gz -h 2019B97.fastp.html -j 2019B97.fastp.json -n 10 -q 20 -u 40
2> 2019B97.fastp.log
```

2．Data Mapping

```
bwa mem -t 5 -R "@RG\tID:2019B94\tSM:2019B94\tPL:ILLUMINA"
Zea_mays.AGPv3.31.dna.genome.fa 2019B97_clean_1.fq.gz 2019B97_clean_2.fq.gz |
samtools   view -b -S -T   Zea_mays.AGPv3.31.dna.genome.fa - > 2019B94.bam
```

3．Filter After Mapping

```
samtools view -h 2019B97.bam   |awk '{if($1~/@/){print}else{if( $7 =="=" &&
$5>=30 ){print $0}}}'   |samtoolsview -b -S -T Zea_mays.AGPv3.31.dna.genome.fa   - >
2019B97.bf.bam
```

4．SNP discovery

```
java -Djava.io.tmpdir=/home/tmp -Xmx15g -jar picard.jar SortSam I=2019B97.bf.bam
O=2019B97.sort.bam SORT_ORDER=coordinate
VALIDATION_STRINGENCY=LENIENT
java -Djava.io.tmpdir=/home/tmp -Xmx15g-jarpicard.jar MarkDuplicates
INPUT=2019B97.sort.bam OUTPUT=2019B97.sort.mdup.bam
METRICS_FILE=2019B97.sort.mdup.metrics CREATE_INDEX=true
VALIDATION_STRINGENCY=LENIENT
samtools index 2019B97.sort.bam
bedtools intersect -a   2019B97.sort.bam -b insert.bed   >2019B97.sort.target.bam
samtools index 2019B97.sort.target.bam
java -Djava.io.tmpdir=/home/tmp   -Xmx12g -jar GenomeAnalysisTK.jar    -T
UnifiedGenotyper   -dcov 1000000 -nt 5 -minIndelFrac 0.15 -glm BOTH -l INFO -R
Zea_mays.AGPv3.31.dna.genome.fa -I 2019B97.sort.target.bam -o   2019B97.vcf
```

5．SNP filter

```
java    -Xmx12g   -jar GenomeAnalysisTK.jar   -T VariantFiltration -R
Zea_mays.AGPv3.31.dna.genome.fa -o 2019B97.filt.vcf –variant 2019B97.vcf --
filterExpression "MQ0 >= 4 && ((MQ0 / (1.0 * DP)) > 0.1) " --filterName
"HARD_TO_VALIDATE"   --filterExpression "DP < 5 || QD < 2" --filterName
"LOW_READ_SUPPORT"
```

Supplemental Tables

Supplemental Table 1. Multiple target mSNP panels (1K to 40K) generated from 40K mSNP mother panel and their high-PIC SNPs, random SNPs, total SNPs and haplotypes, evaluated with the marker data of 867 maize germplasm accessions

SNPs derived from 40K mSNPs		40K	20K	10K	5K	2K	1K
**High-PIC SNPs**	Marker #	38,034	21,677	10,085	5,000	2,000	1,000
	MAF>5%	33,080	18,745	8,702	4,389	1,769	884
**Random SNPs**	Marker #	38,034	21,677	10,085	5,000	2,000	1,000
	MAF>5%	35,837	20,437	9,488	4726	1,893	947
**Total SNPs**	Marker #	251,630	148,936	69,327	33,711	13,101	6,493
	MAF>5%	236,210	139,964	65,096	31,813	12,378	6,120
**Haplotypes**	Marker #	690,056	412,396	194,629	92,937	36,882	18,370
	MAF>5%	159,255	92,061	43,491	21,642	8,605	4,294

Supplemental Table 2. Genetic variation detected among the maize groups with 867 maize inbred lines

Maize groups	Sample size	mSNP	SNP# MAF>5%	Haplotype (realized)	SNP# /mSNP	Haplptype /mSNP	PIC-SNP	PIC-Haplotype
Temperate	325	38,022	234,391	333,587	6.62	8.77	0.272	0.610
Iodent	32	37,708	200,244	158,093	6.64	4.19	0.223	0.486
Lancaster	64	37,977	209,039	200,474	6.62	5.28	0.228	0.501
LRC	37	37,922	221,404	192,361	6.63	5.07	0.245	0.554
PA	40	37,653	179,221	159,995	6.65	4.25	0.203	0.436
PB	36	37,785	200,445	181,408	6.64	4.80	0.221	0.500
Reid	60	37,828	182,734	176,127	6.63	4.66	0.207	0.444
SPT	56	37,957	217,471	204,281	6.62	5.38	0.236	0.535
Trop	288	38,019	216,176	402,015	6.62	10.57	0.253	0.605
Sweet	246	37,934	194,115	453,978	6.62	11.97	0.226	0.582

Heterotic groups in temperate maize: Iodent, Lancaster, LRC, PA, PB, Reid and SPT

Supplemental Table 3. 40K mSNPs, 251 SNPs and 159K haplotypes identified in different genomic regions using 867 maize inbred lines (CDS = exon)

Marker types		UTR5	Intergenic	CDS	UTR3	Intronic	Total
40K high-PIC	#	644	28544	1420	1477	5949	38034
SNPs	%	1.69	75.05	3.73	3.88	15.64	
251K SNPs	#	3585	194198	7794	8930	37124	251630
	%	1.42	77.18	3.10	3.55	14.75	
159K Haplotypes	#	3202	114155	8568	7245	26085	159255
	%	2.01	71.68	5.38	4.55	16.38	

Supplemental Table 4. Distribution of numbers and frequencies of insertion and deletion identified with 251K SNPs in genic and intergenic regions

	UTR5	Intergenic	CDS	UTR3	Intronic	Total
Insertion	40 (1.12)	1678 (0.86)	35 (0.45)	87 (0.97)	327 (0.88)	2167
Deletion	53 (1.48)	2893 (1.49)	34 (0.44)	133 (1.49)	520 (1.40)	3633
Total	93 (2.59)	4571 (2.35)	69 (0.89)	220 (2.46)	847 (2.28)	5800

The numbers in the parentheses are frequencies (%), calculated by comparing the number of insertion or deletion with the total number of SNPs identified in the same genomic region as shown in Supplemental Table 3.

Supplemental Table 5. LD decay distances estimated by different types of markers from genic and intergenic regions with different germplasm groups

**Germplasm**	**Marker types**	**Genomic regions**	**Decay distance (Kb)**	
			**r^2=0.1**	**r^2=0.2**
All germplasm	251K SNPs	Genic	12	2
		Intergenic	73	5
		Total	40	3
	40K high-PIC SNPs	Genic	26	9
		Intergenic	149	39
		Total	114	31
Sweet	251K SNPs	Genic	33	5
		Intergenic	195	7
		Total	180	5
	40K high-PIC SNPs	Genic	110	18
		Intergenic	499	95
		Total	480	79
Temperate	251K SNPs	Genic	29	4
		Intergenic	170	6
		Total	129	4
	40K high-PIC SNPs	Genic	38	13
		Intergenic	295	88
		Total	306	57
Tropical	251K SNPs	Genic	7	2
		Intergenic	38	3
		Total	26	3
	40K high-PIC SNPs	Genic	21	5
		Intergenic	89	36
		Total	80	7

Supplemental Figures

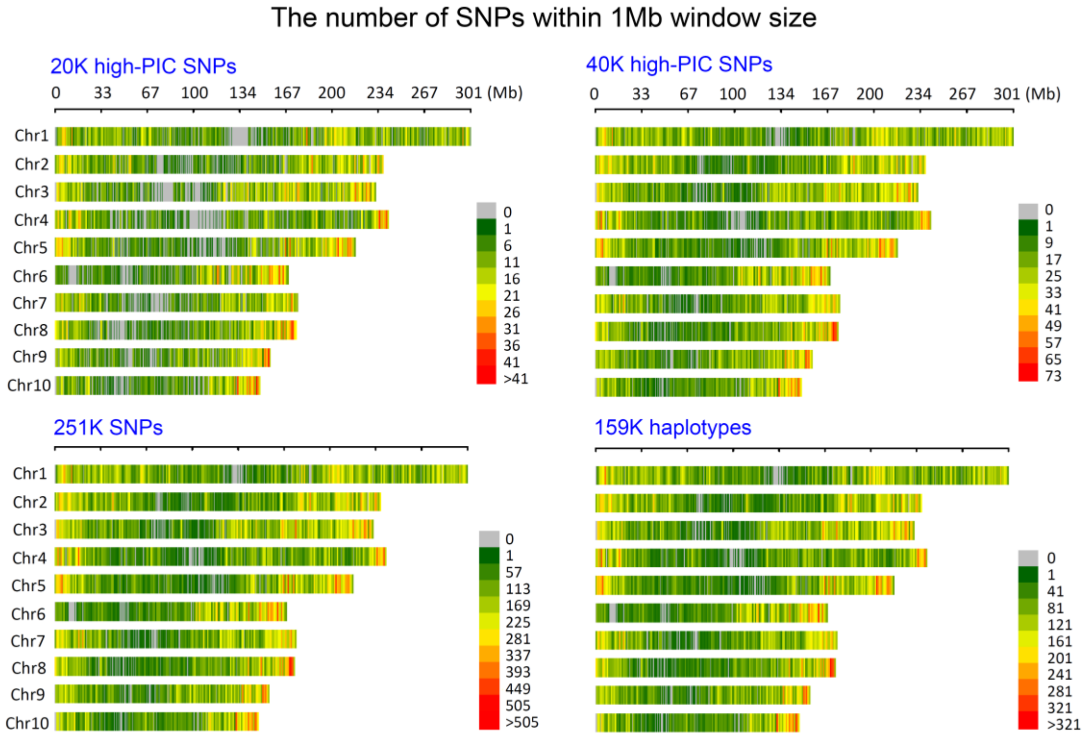

Supplemental Figure 1. Distribution of GBTS markers on maize chromosomes. Chromosome distribution was provided for 20K high-PIC SNPs, 40K high-PIC SNPs, 251K SNPs and 159K haplotypes. Marker density is indicated by bar color, and each bar represents 1-Mb window.

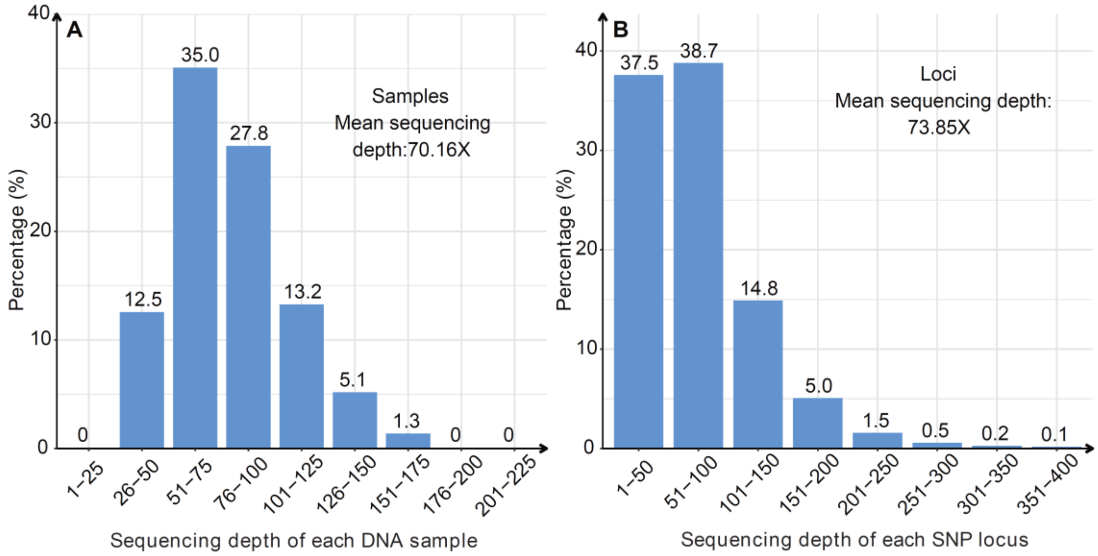

Supplemental Figure 2. Sequencing depths for genotyping. Distribution of sequencing depths for samples (867 maize inbred lines; A) and loci (40K mSNP loci; B).

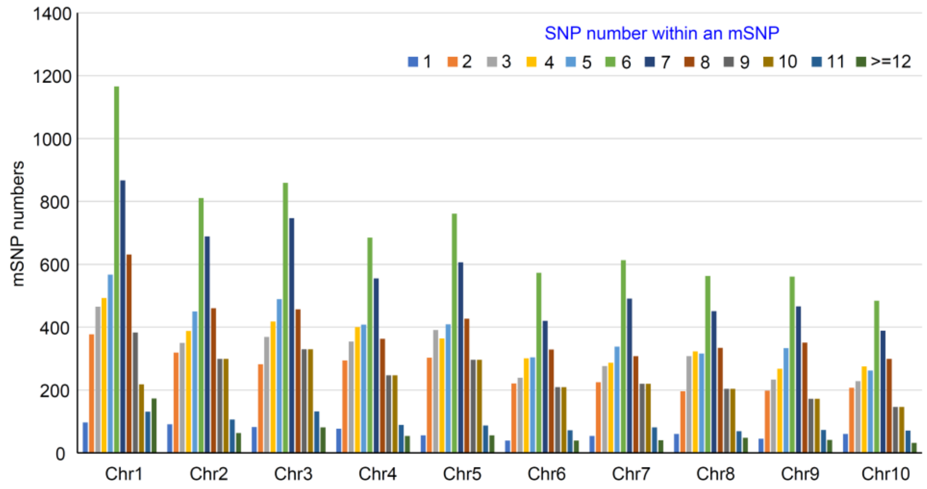

Supplemental Figure 3. Frequency distribution of 40K mSNPs on chromosomes. The numbers of the SNPs covered by mSNPs are coded by different colors.

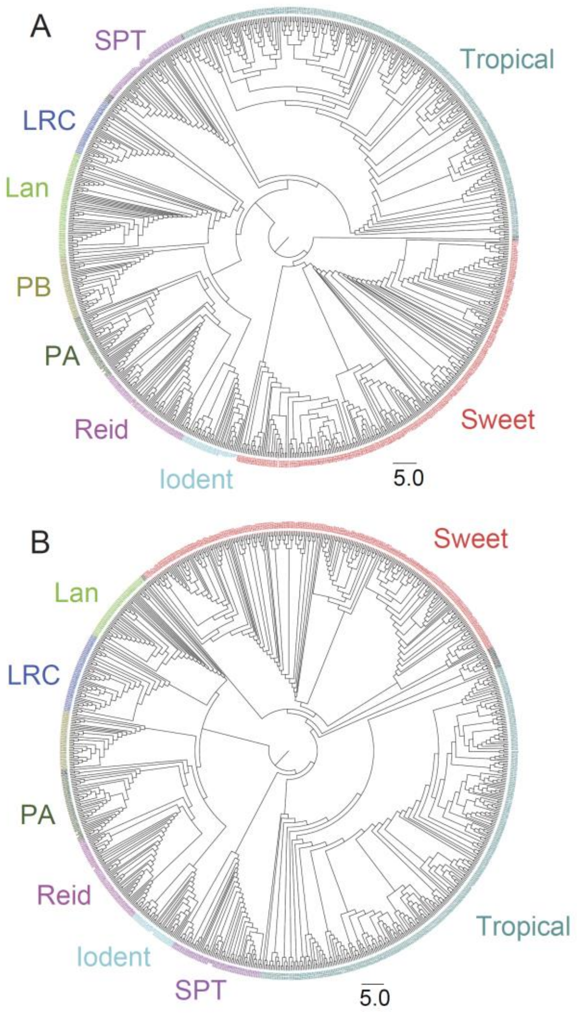

Supplemental Figure 4. Evaluation of markers by phylogenetic analysis. Phylogenetic trees constructed using maize inbred lines for the germplasm sample combined (n=867) using UPGMA and two marker types, 40K high-PIC SNPs (A) and 251K SNPs (B). Identical groups in the two trees can be identified by flipping the relevant tree branches.

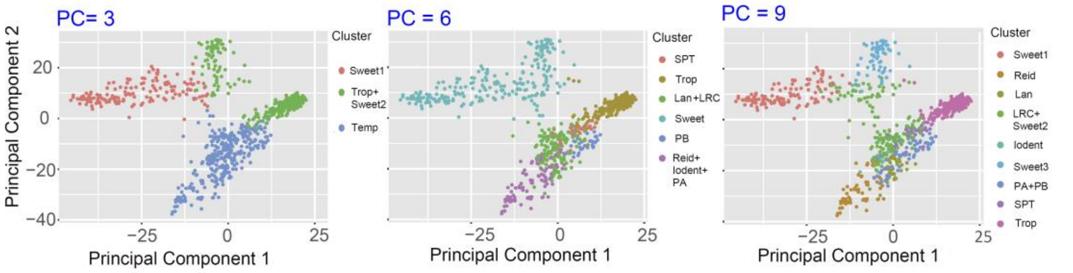

Supplemental Figure 5. Evaluation of markers by principal component analysis. Pairwise Modified Rogers' distances were estimated for 867 maize inbred lines using 40K high-PIC SNPs.

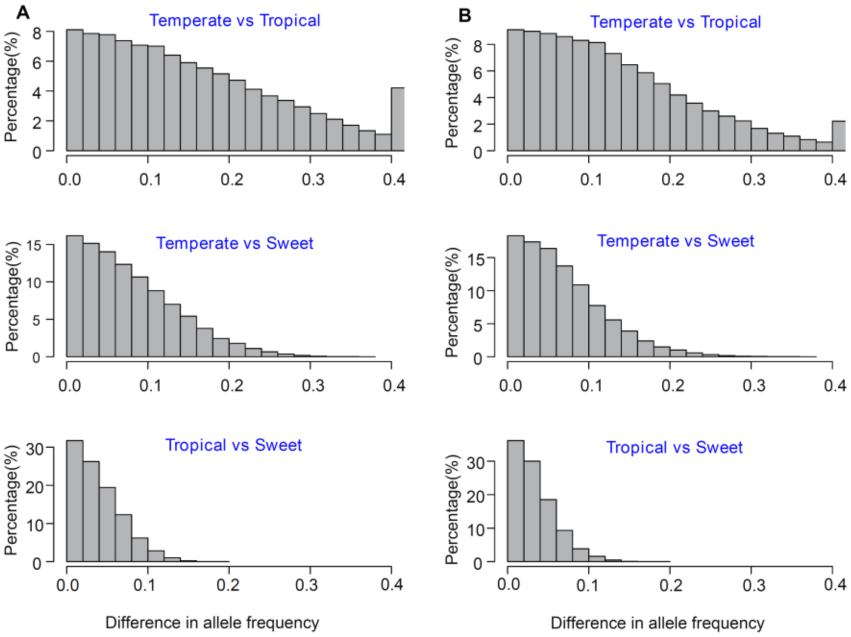

Supplemental Figure 6. Differentiation of allele frequencies between maize groups. The groups of 867 maize germplasm accessions were revealed by 40K high-PIC SNPs (A) and 251K SNPs (B). Comparisons between three germplasm groups: Temperate versus Tropical, Temperate versus Sweet, and Tropical versus Sweet.

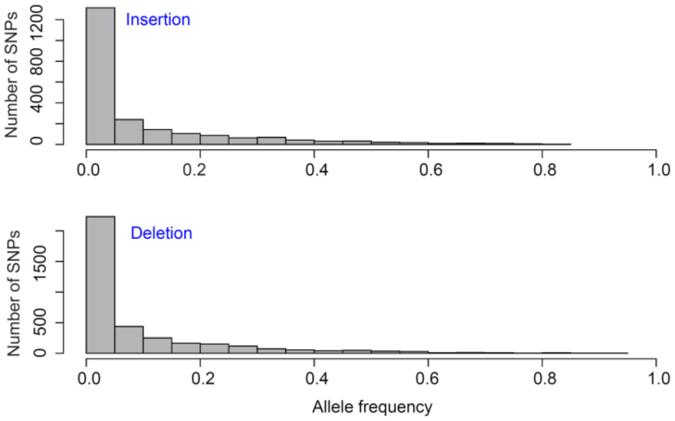

Supplemental Figure 7. Distribution of frequencies for insertions and deletions. A total of 251K SNPs were used for calculation of allele frequencies.
